# Supplementary material for: Identifying critical features of iron phosphate particle for lithium preference
Source: Nat Commun. 2024 Jun 7;15:4859. doi: 10.1038/s41467-024-49191-3 (PMC11161493; doi:10.1038/s41467-024-49191-3)
Supplement: Supplementary file 1 — Supplementary Information [file 41467_2024_49191_MOESM1_ESM.pdf]

Supplementary Information

Identifying critical features of iron phosphate particle for lithium preference

Gangbin Yan[1], Jialiang Wei[2], Emory Apodaca[1], Suin Choi[1], Peter J. Eng[3,4], Joanne E. Stubbs[3], Yu Han[1], Siqi Zou[1], Mrinal K. Bera[5], Ronghui Wu[1], Evguenia Karapetrova[6], Hua Zhou[6], Wei Chen[2,7] and Chong Liu[1,*]

[1]Pritzker School of Molecular Engineering, University of Chicago, Chicago, IL 60637, USA
[2]Department of Mechanical, Materials and Aerospace Engineering, Illinois Institute of Technology, Chicago, IL 60616, USA
[3]Center for Advanced Radiation Sources, University of Chicago, Chicago, IL 60637, USA
[4]James Frank Institute, University of Chicago, Chicago, IL 60637, USA
[5]NSF's ChemMatCARS, Pritzker School of Molecular Engineering, University of Chicago, Chicago, IL 60637, USA
[6]X-Ray Science Division, Advanced Photon Source, Argonne National Laboratory, Lemont, IL 60439, USA
[7]Department of Materials Design and Innovation, University at Buffalo, The State University of New York, Buffalo, NY, 14260, USA
[*]Correspondence: chongliu@uchicago.edu

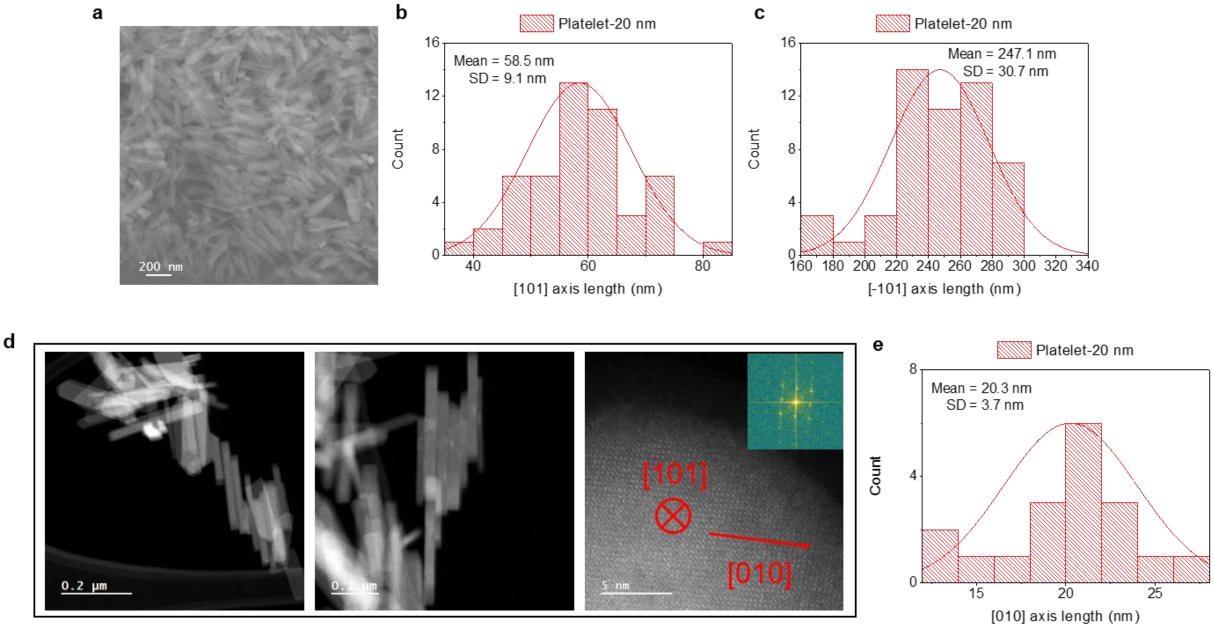

Supplementary Figure 1 Synthesized Platelet-20 nm $LiFePO_4$ particles and summarized particle dimensions. (a) SEM images. (b) Particle dimension distribution along the [101] axis. (c) Particle dimension distribution along the [-101] axis. (d) STEM images. The inset figure on the right shows the Fast Fourier Transform (FFT) of the corresponding high-resolution image. (e) Particle dimension distribution along the [010] axis. (Only particles with fully exposed dimensions were counted.)

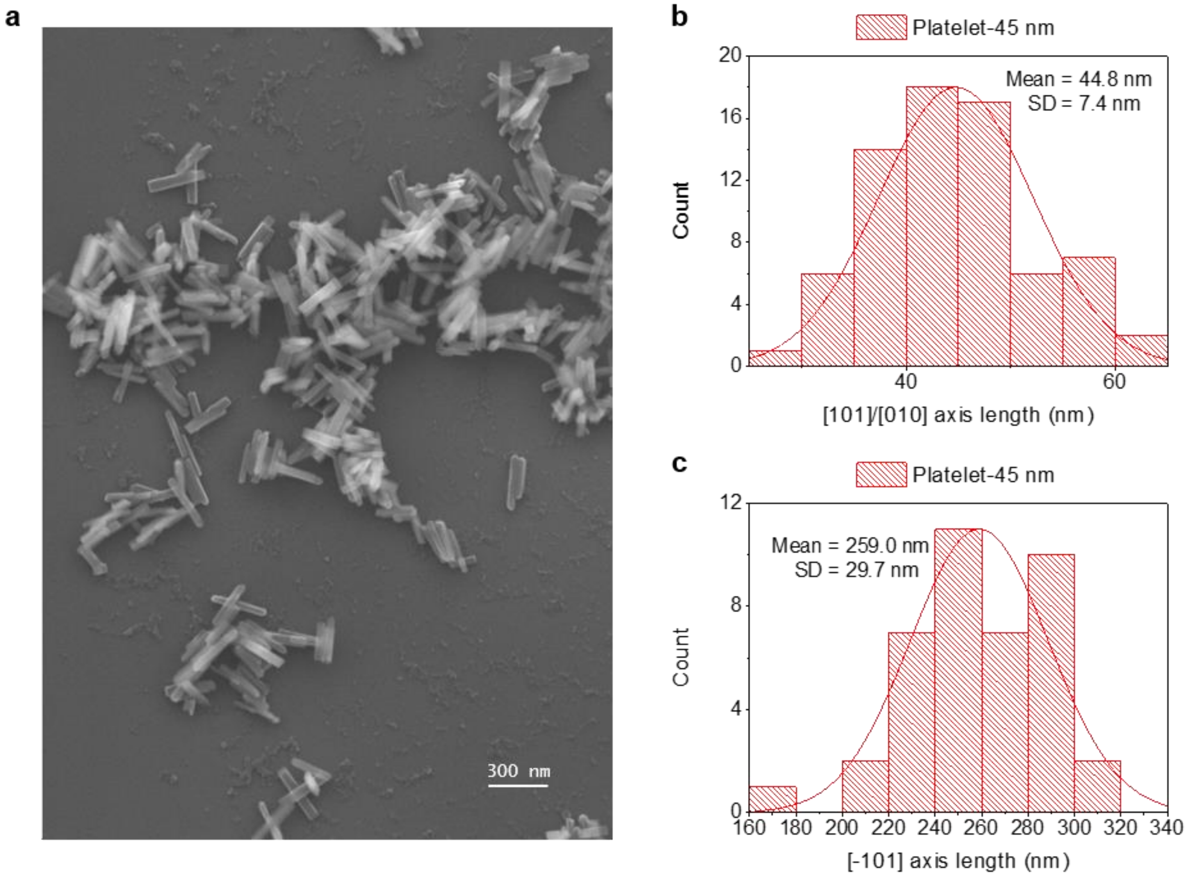

Supplementary Figure 2 Synthesized Platelet-45 nm $LiFePO_4$ particles and summarized particle dimensions. (a) SEM images. (b) Particle dimension distribution along the [101]/[010] axis. (c) Particle dimension distribution along the [-101] axis. (Only particles with fully exposed dimensions were counted.)

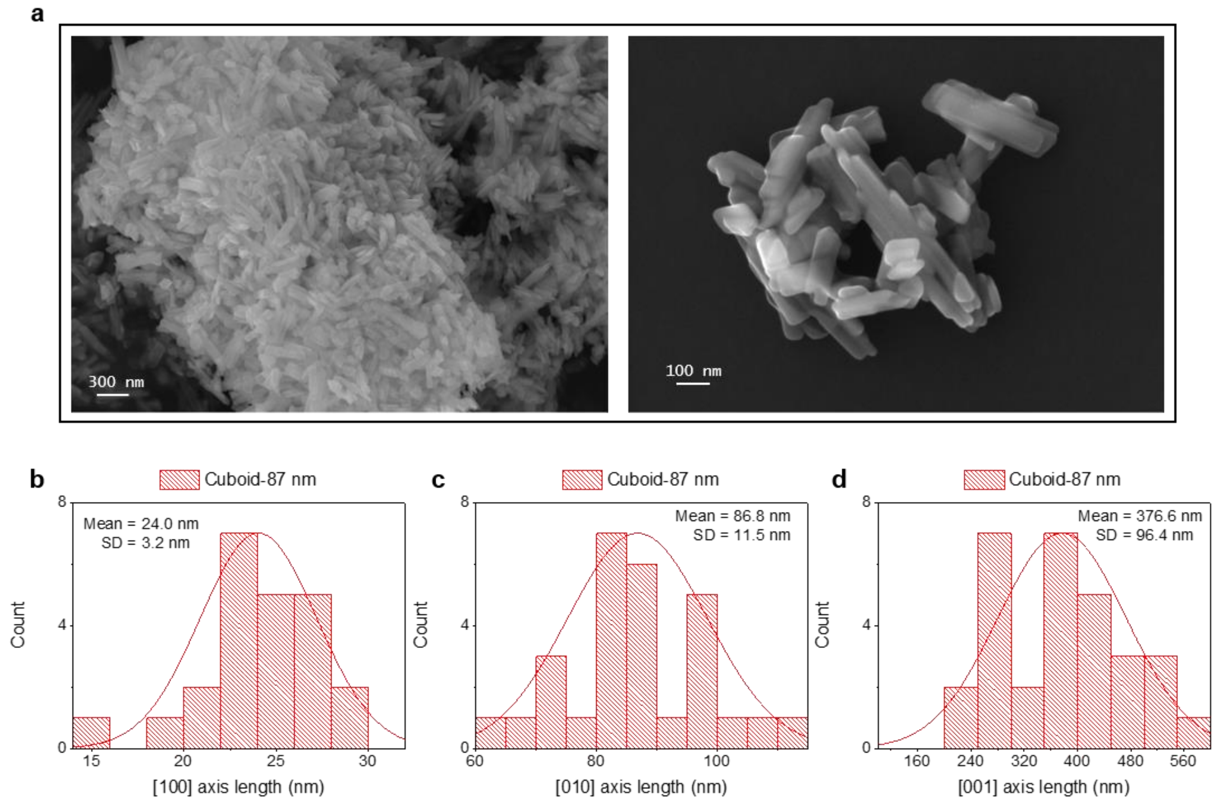

Supplementary Figure 3 Synthesized Cuboid-87 nm $LiFePO_4$ particles and summarized particle dimensions. (a) SEM images. (b) Particle dimension distribution along the [100] axis. (c) Particle dimension distribution along the [010] axis. (d) Particle dimension distribution along the [001] axis. (Only particles with fully exposed dimensions were counted.)

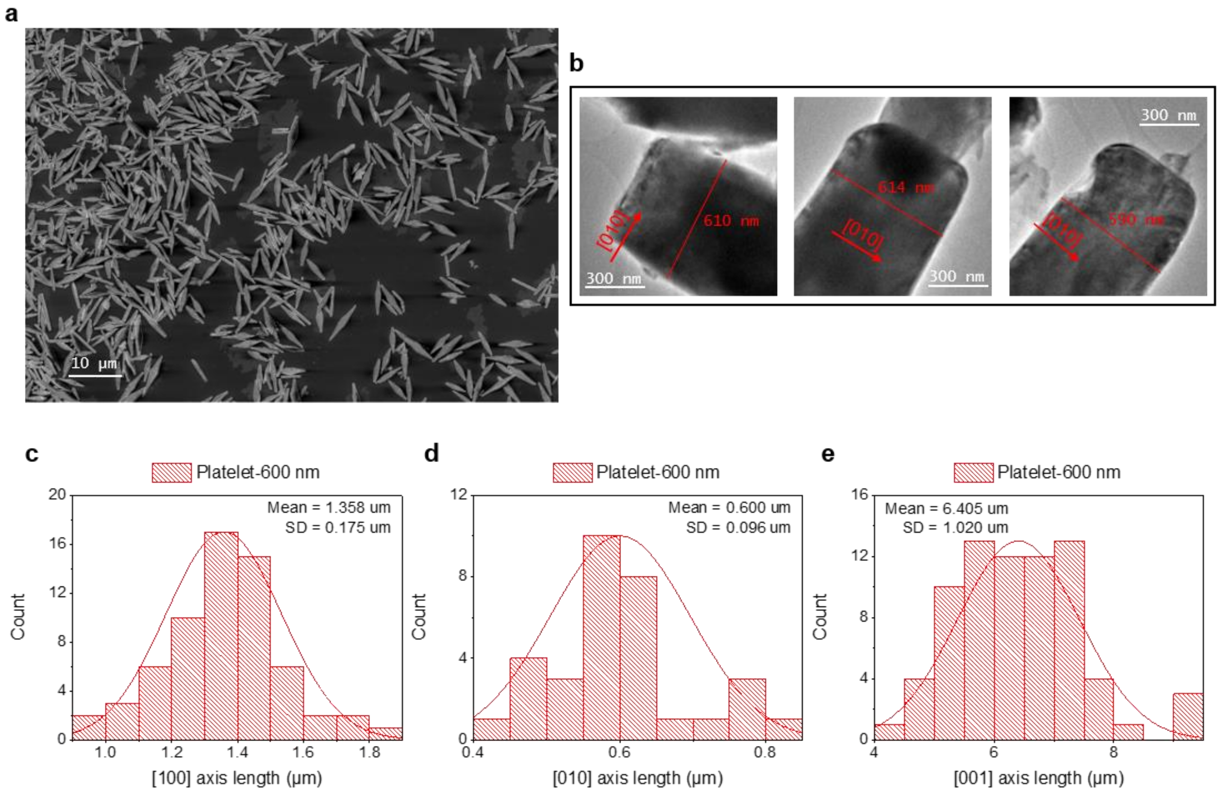

Supplementary Figure 4 Synthesized Platelet-600 nm $LiFePO_4$ particles and summarized particle dimensions. (a) SEM images. (b) STEM images. (c) Particle dimension distribution along the [100] axis. (d) Particle dimension distribution along the [010] axis. (e) Particle dimension distribution along the [001] axis. (Only particles with fully exposed dimensions were counted.)

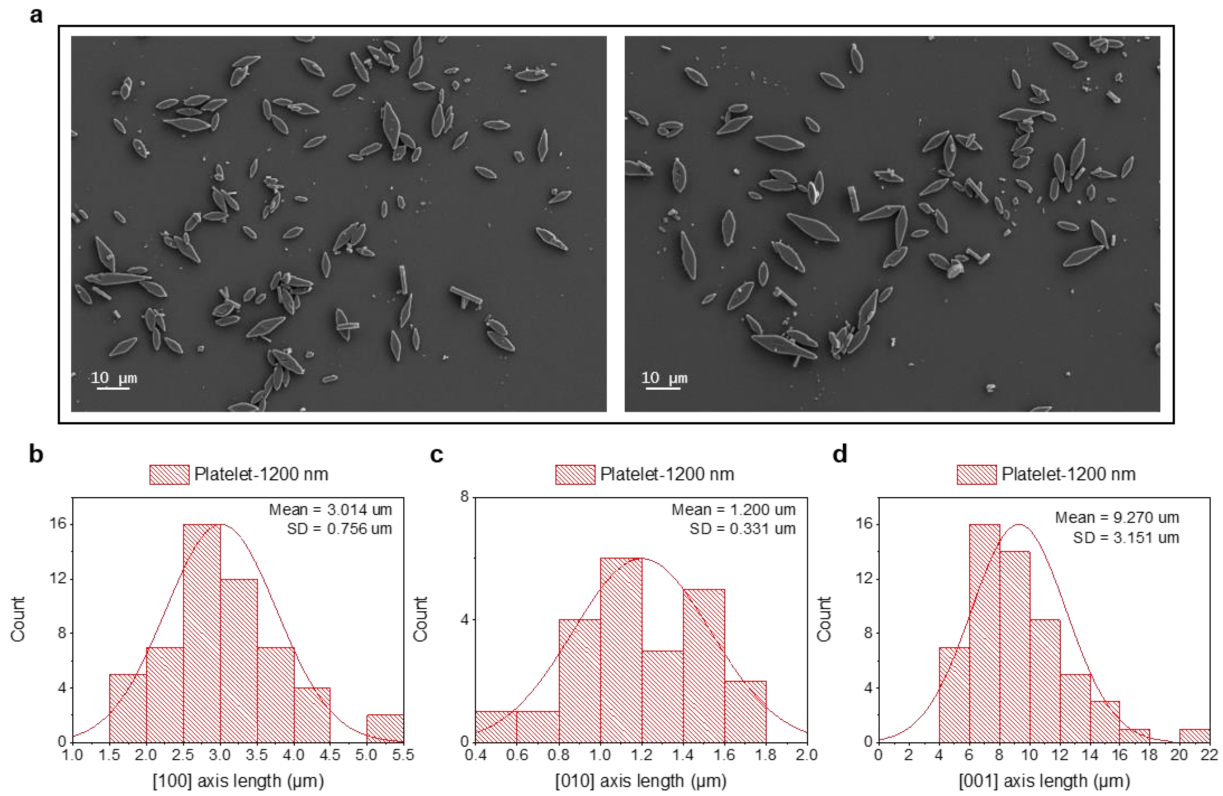

Supplementary Figure 5 Synthesized Platelet-1200 nm $LiFePO_4$ particles and summarized particle dimensions. (a) SEM images. (b) Particle dimension distribution along the [100] axis. (c) Particle dimension distribution along the [010] axis. (d) Particle dimension distribution along the [001] axis. (Only particles with fully exposed dimensions were counted.)

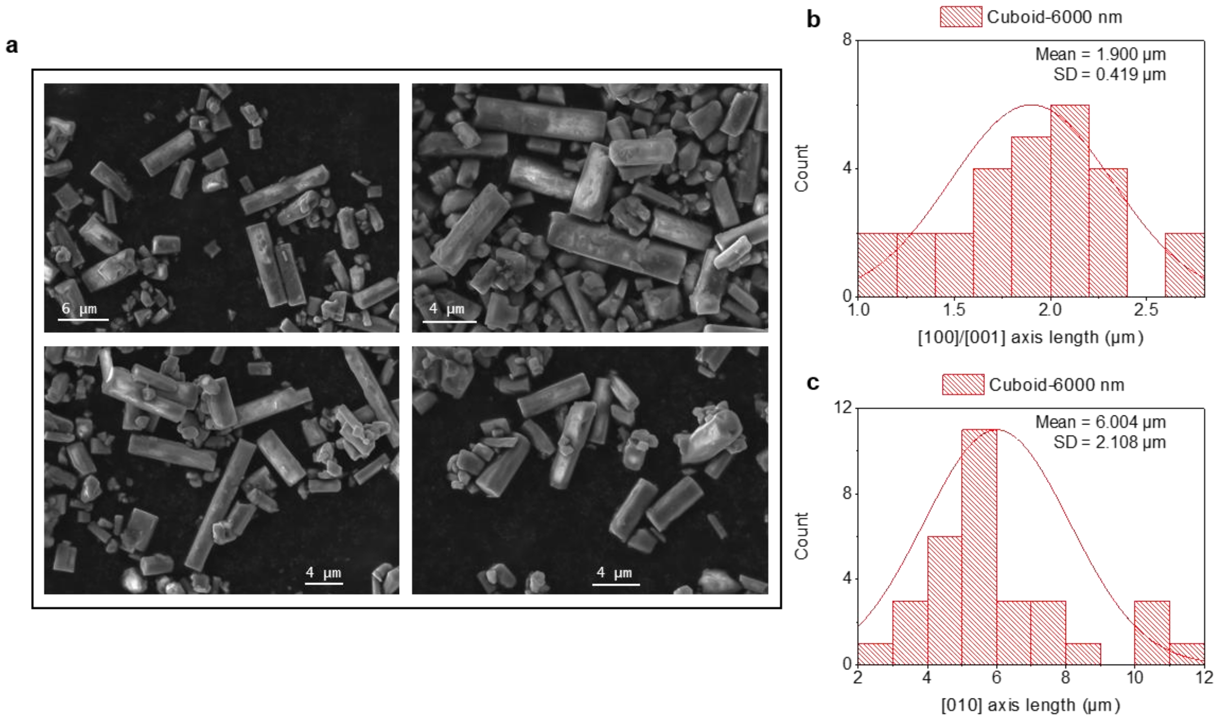

Supplementary Figure 6 Synthesized Cuboid-6000 nm $LiFePO_4$ particles and summarized particle dimensions. (a) SEM images. (b) Particle dimension distribution along the [100]/[001] axis. (c) Particle dimension distribution along the [010] axis. (Only particles with fully exposed dimensions were counted.)

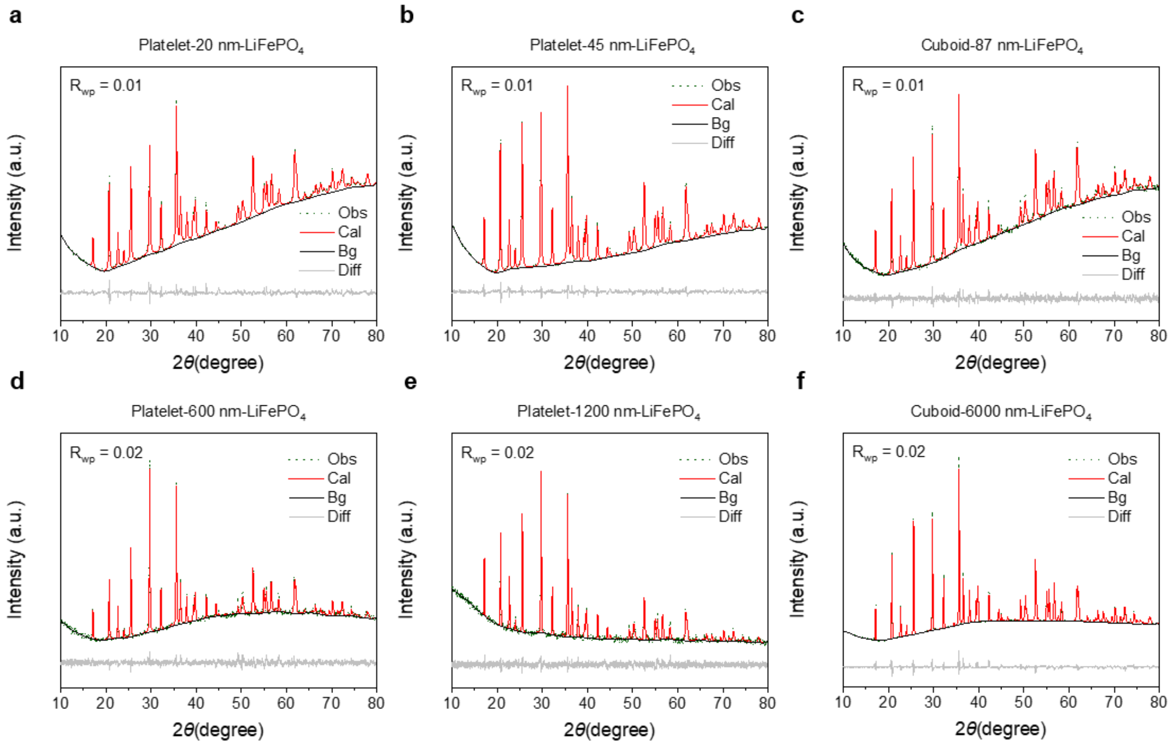

Supplementary Figure 7 Rietveld refinement of $LiFePO_4$ particles (a) Platelet-20 nm, (b) Platelet-45 nm, (c) Cuboid-87 nm, (d) Platelet-600 nm, (e) Platelet-1200 nm, and (f) Cuboid-6000 nm.

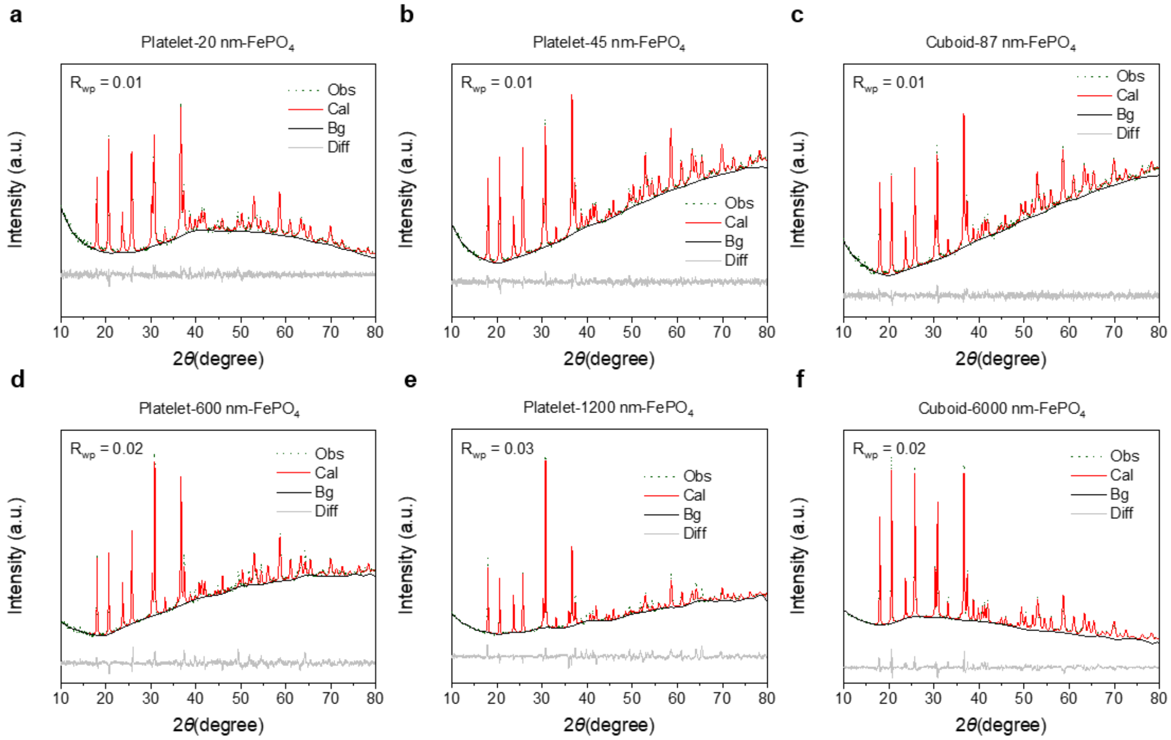

Supplementary Figure 8 Rietveld refinement of $FePO_4$ particles (a) Platelet-20 nm, (b) Platelet-45 nm, (c) Cuboid-87 nm, (d) Platelet-600 nm, (e) Platelet-1200 nm, and (f) Cuboid-6000 nm.

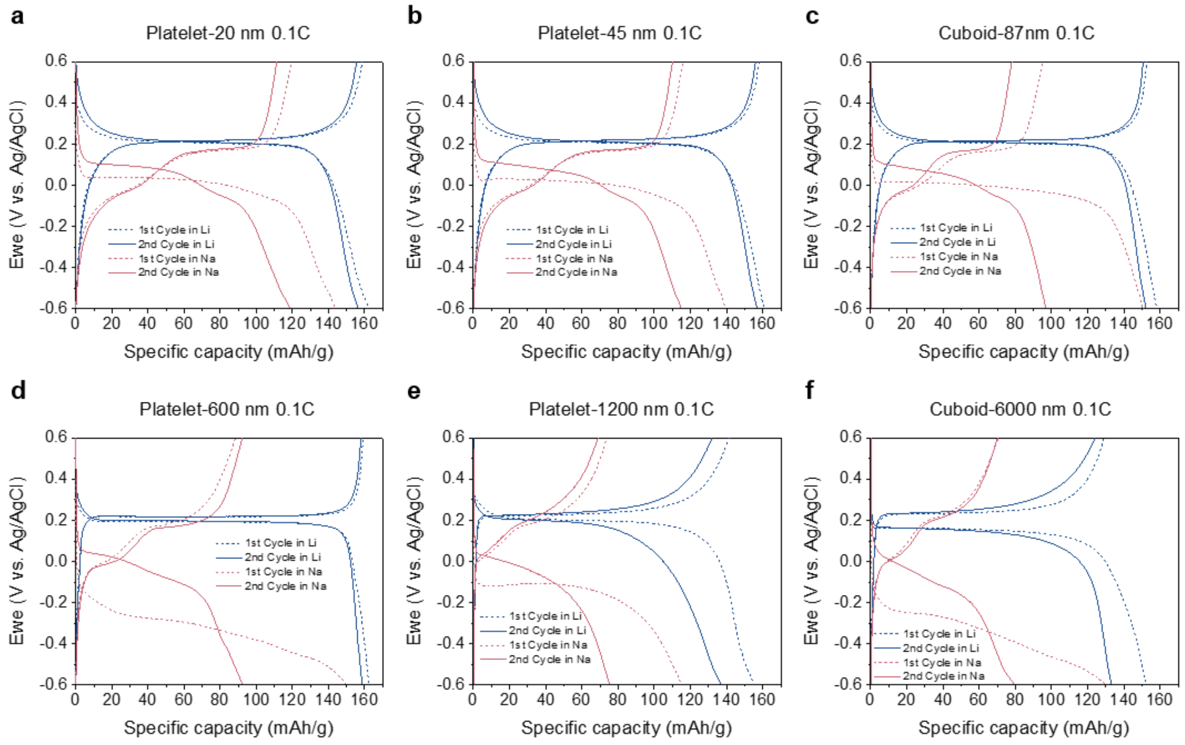

Supplementary Figure 9 Electrochemical cycling of the chemically extracted $FePO_4$ electrodes in either 60 mL 1 M LiCl aqueous solutions (17 mA/g; paired with Li_xFePO_4 counter electrodes) or 60 mL 1 M NaCl aqueous solutions (15.4 mA/g; paired with Na_yFePO_4 counter electrodes) between - 0.6 V and 0.6 V (vs. Ag/AgCl/KCl (4.0 M)) at room temperature (20 ~ 25 °C). (a) Platelet-20 nm, (b) Platelet-45 nm, (c) Cuboid-87 nm, (d) Platelet-600 nm, (e) Platelet-1200 nm, and (f) Cuboid-6000 nm. N_2 (purity > 99.998%) was continuously bubbled into the solution to avoid side reactions caused from dissolved O_2.

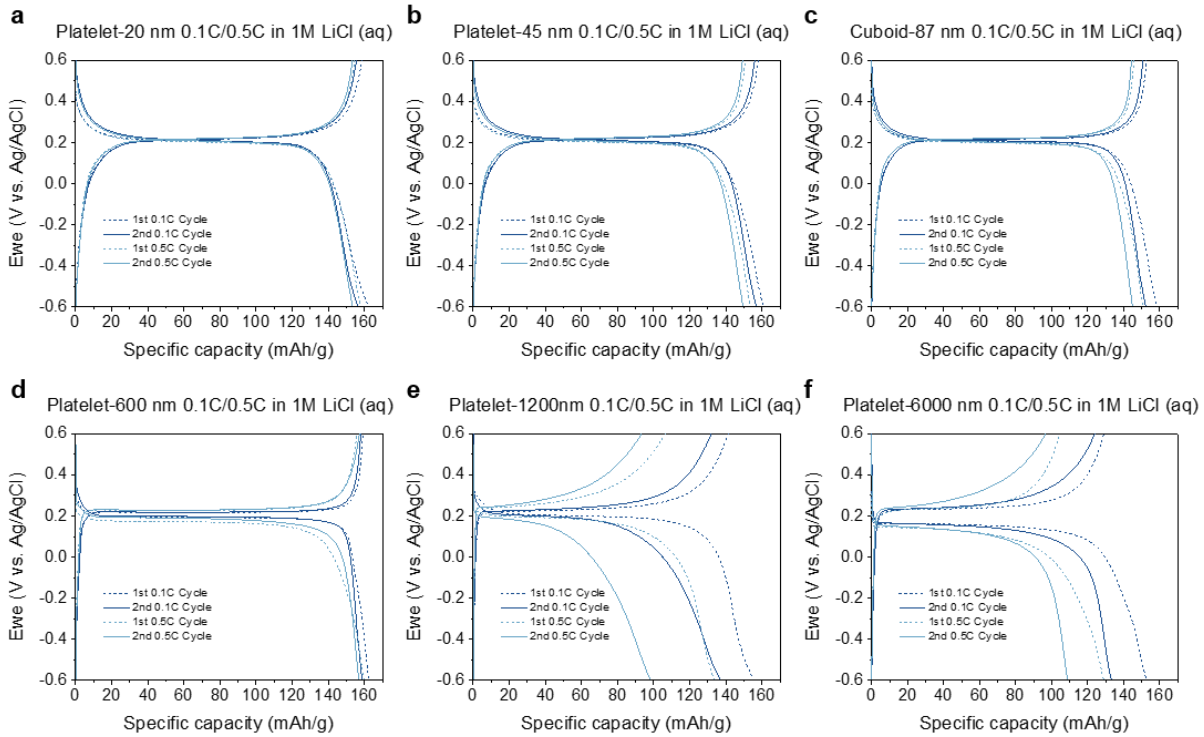

Supplementary Figure 10 Electrochemical cycling of the chemically extracted $FePO_4$ electrodes at 0.1C (17 mA/g) or 0.5C (85 mA/g) in 60 mL 1 M LiCl aqueous solutions (Paired with Li_xFePO_4 counter electrodes) between - 0.6 V and 0.6 V (vs. Ag/AgCl/KCl (4.0 M)) at room temperature (20 ~ 25 °C). (a) Platelet-20 nm, (b) Platelet-45 nm, (c) Cuboid-87 nm, (d) Platelet-600 nm, (e) Platelet-1200 nm, and (f) Cuboid-6000 nm. N_2 (purity > 99.998%) was continuously bubbled into the solution to avoid side reactions caused from dissolved O_2.

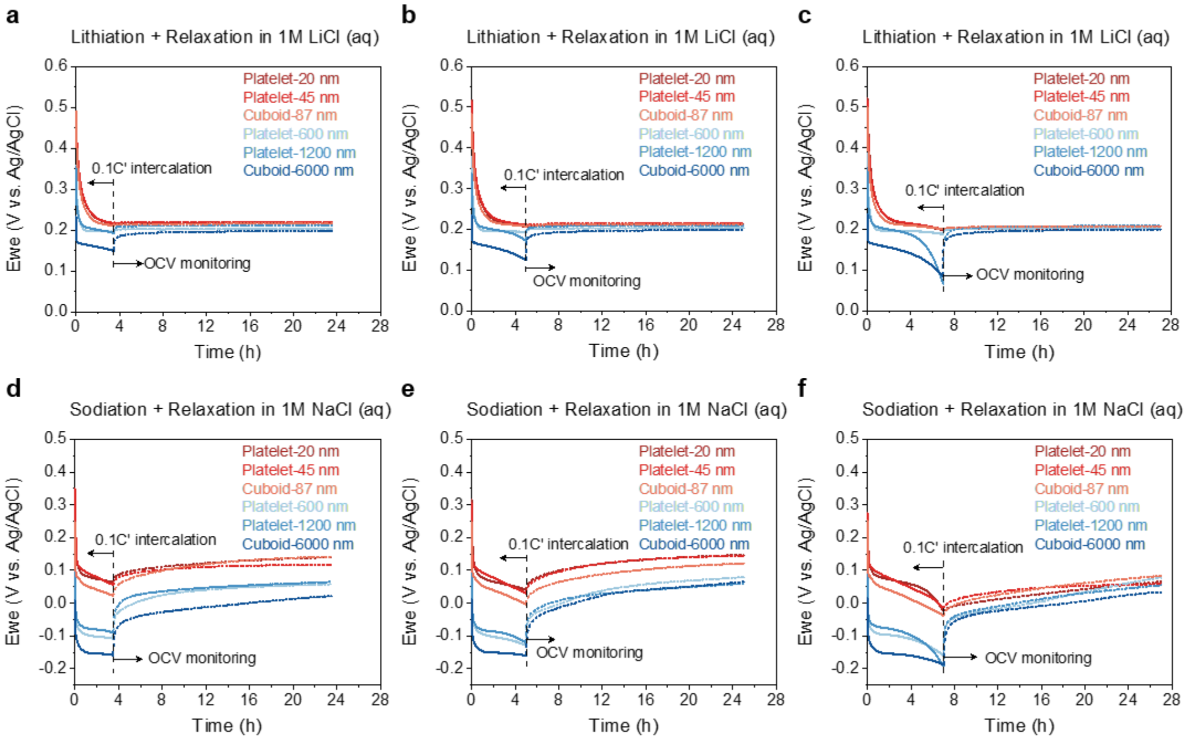

Supplementary Figure 11 Intercalation (0.1C') until different depth-of-discharge (DOD) (Li/Na_0.35'/0.5'/0.7') followed by 20 hours open circuit voltage (OCV) monitoring in the original solutions. Electrodes are precycled in 1 M LiCl aqueous solutions to measure the accessible capacity (See Methods for more details). The calculations of applied current and depth of discharge are both based on the delivered capacity in the first de-lithiation instead of the theoretical capacity. To differentiate the C-rates calculated using different approaches, we denote the C-rates here as C' instead of C. For instance, 0.1C' for the Platelet-20 nm particle will be 15.9 mA/g (Supplementary Table 5). For lithiation, 60 mL 1 M LiCl aqueous solutions were used, paired with Li_xFePO_4 counter electrodes operating at room temperature (20 ~ 25 °C); for sodiation, 60 mL 1 M NaCl aqueous solutions were used, paired with Na_yFePO_4 counter electrodes operating at room temperature (20 ~ 25 °C). N_2 (purity > 99.998%) was continuously bubbled into the solution to avoid side reactions caused from dissolved O_2. (a) Lithiation until 35% accessible capacity then OCV monitoring. (b) Lithiation until 50% accessible capacity then OCV monitoring. (c) Lithiation until 70% accessible capacity then OCV monitoring. (d) Sodiation until 35% accessible capacity then OCV monitoring. (e) Sodiation until 50% accessible capacity then OCV monitoring. (f) Sodiation until 70% accessible capacity then OCV monitoring.

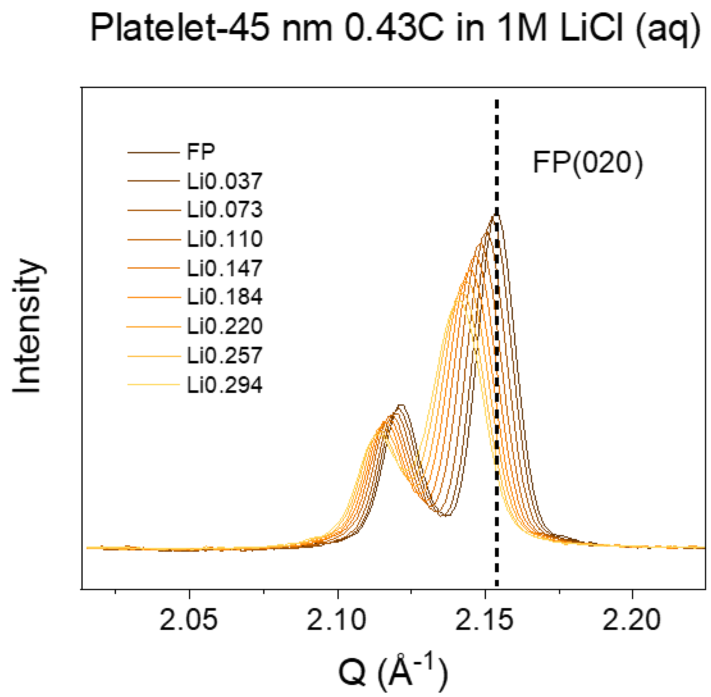

Supplementary Figure 12 *In situ* synchrotron XRD tracking of Platelet-45 nm particles during 0.43C (based on theoretical capacity, 1C is equivalent to 170 mA/g) lithiation. Before *in situ* experiments, electrodes are precycled in 1 M LiCl aqueous solutions to measure the accessible capacity and verify the *in situ* setup. Continuous change of peak positions is witnessed, which shows a SS phase evolution.

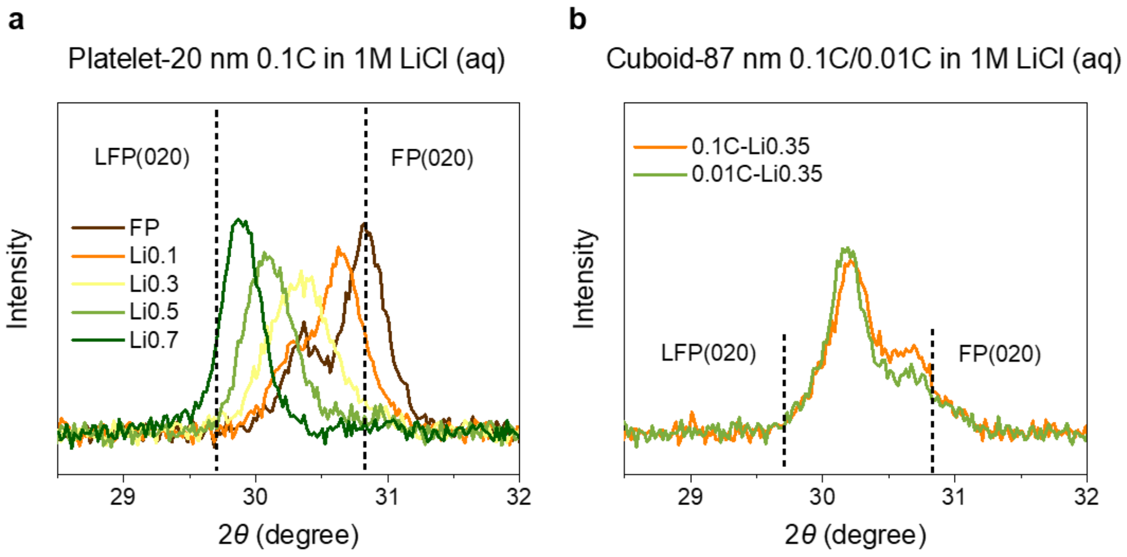

Supplementary Figure 13 *Ex situ* in-house XRD (λ = 1.54 Å) of electrodes at different depth-of-lithiation using 0.1C/0.01C (1C is equivalent to 170 mA/g). (a) Platelet-20 nm particles until 10%/30%/50%/70% theoretical capacity usage prepared at 0.1C. (b) Cuboid-87 nm particles until 35% theoretical capacity usage prepared at 0.1C/0.01C.

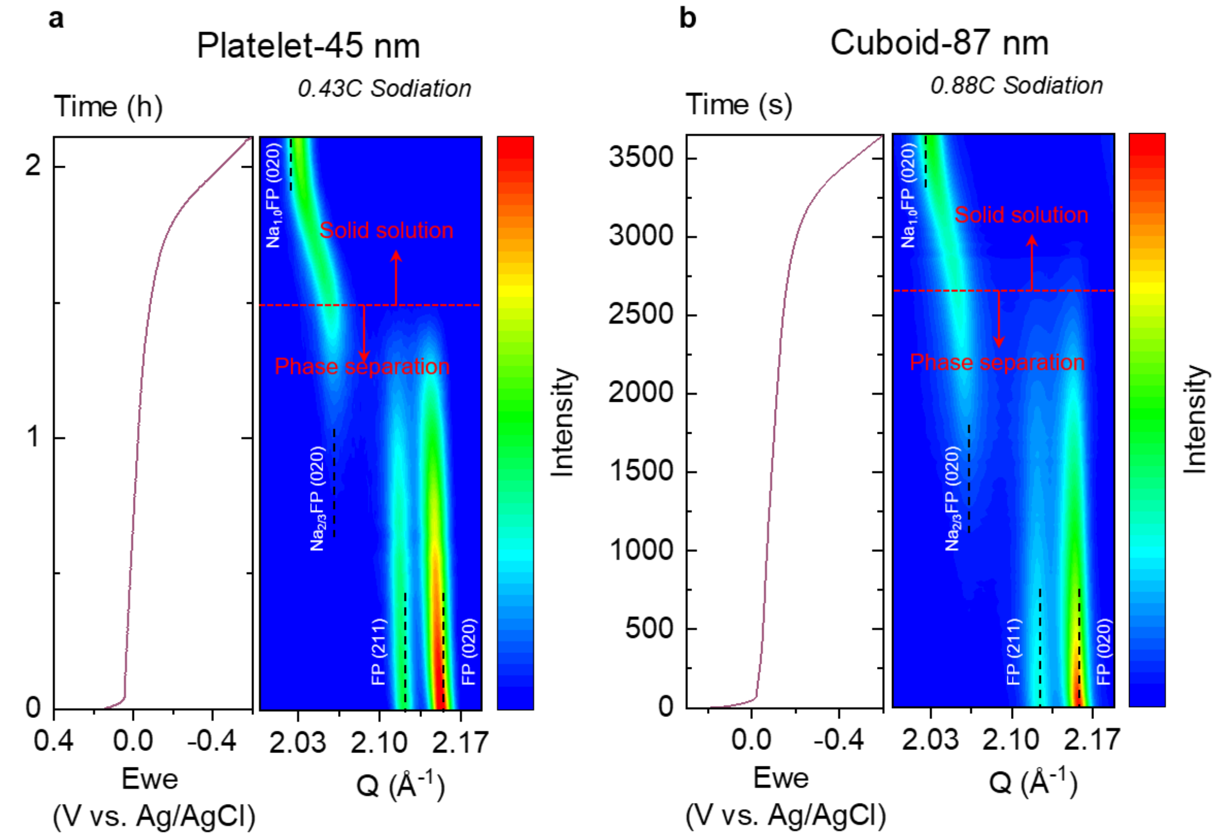

Supplementary Figure 14 *In situ* synchrotron XRD tracking of phase evolutions during sodiation. For sodiation, 1C is equivalent to 154 mA/g. (a) Sodiation of Platelet-45 nm particles at 0.43C. (b) Sodiation of Cuboid-87 nm particles at 0.88C.

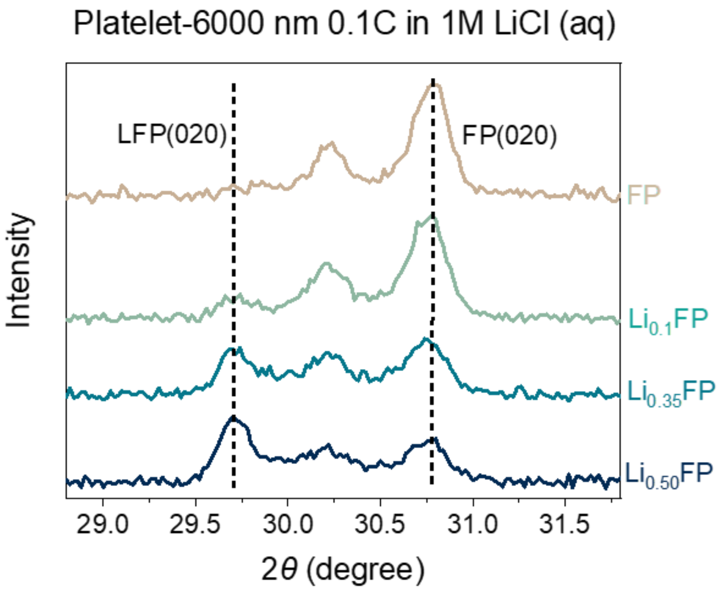

Supplementary Figure 15 ***Ex situ*** **in-house XRD (λ = 1.54 Å) of Platelet-6000 nm particles at different depth-of-lithiation (FP/Li0.1FP/Li0.35FP/Li0.5FP) prepared with 0.1C (1C is equivalent to 170 mA/g).**

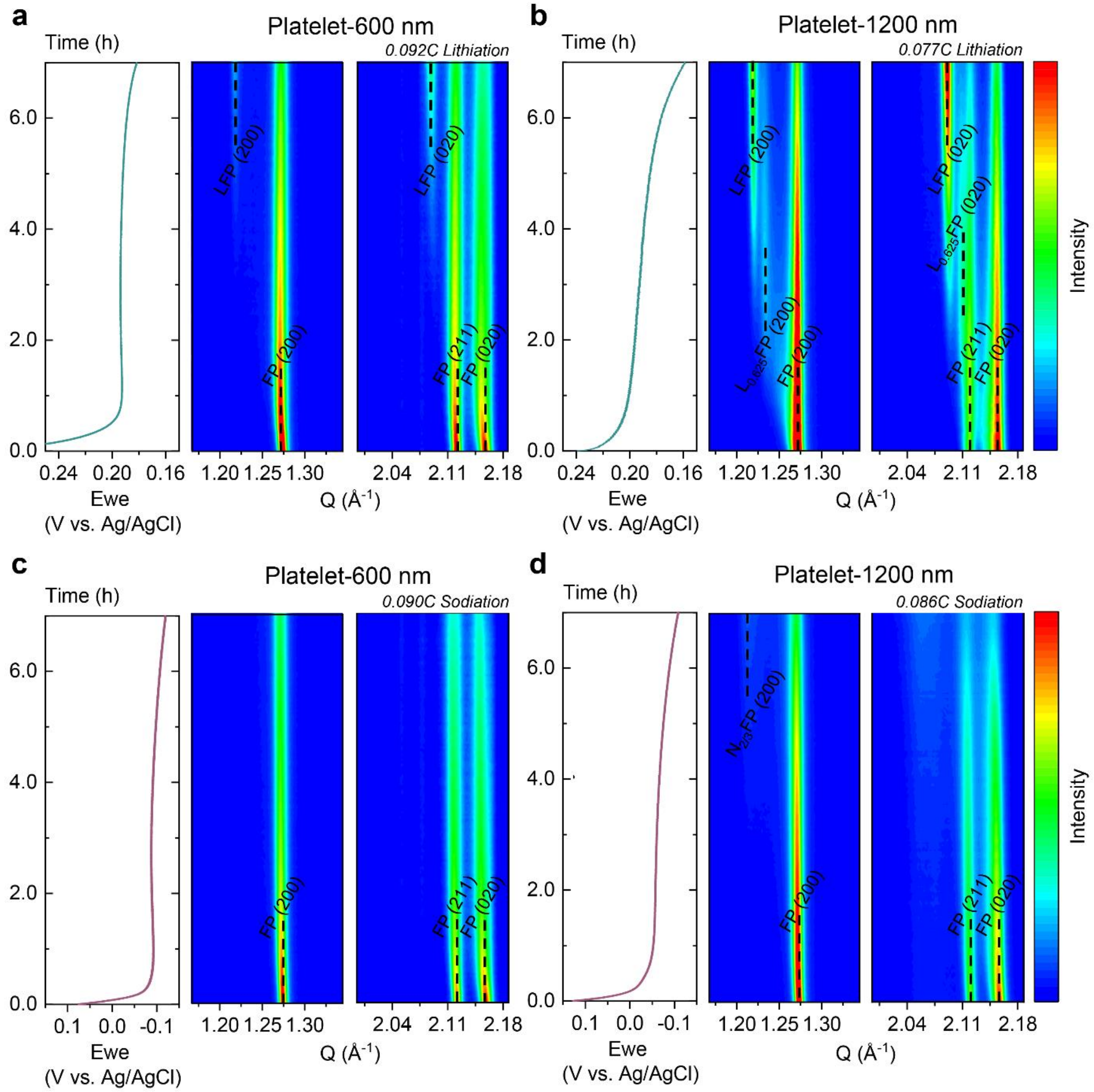

Supplementary Figure 16 ***In situ*** **synchrotron XRD tracking of phase evolutions during lithiation or sodiation.** For lithiation, 1C is equivalent to 170 mA/g, while for sodiation, 1C is equivalent to 154 mA/g. (a) Lithiation of Platelet-600 nm particles at 0.092C. (b) Lithiation of Platelet-1200 nm particles at 0.077C. (c) Sodiation of Platelet-600 nm particles at 0.090C. (d) Sodiation of Platelet-1200 nm particles at 0.086C.

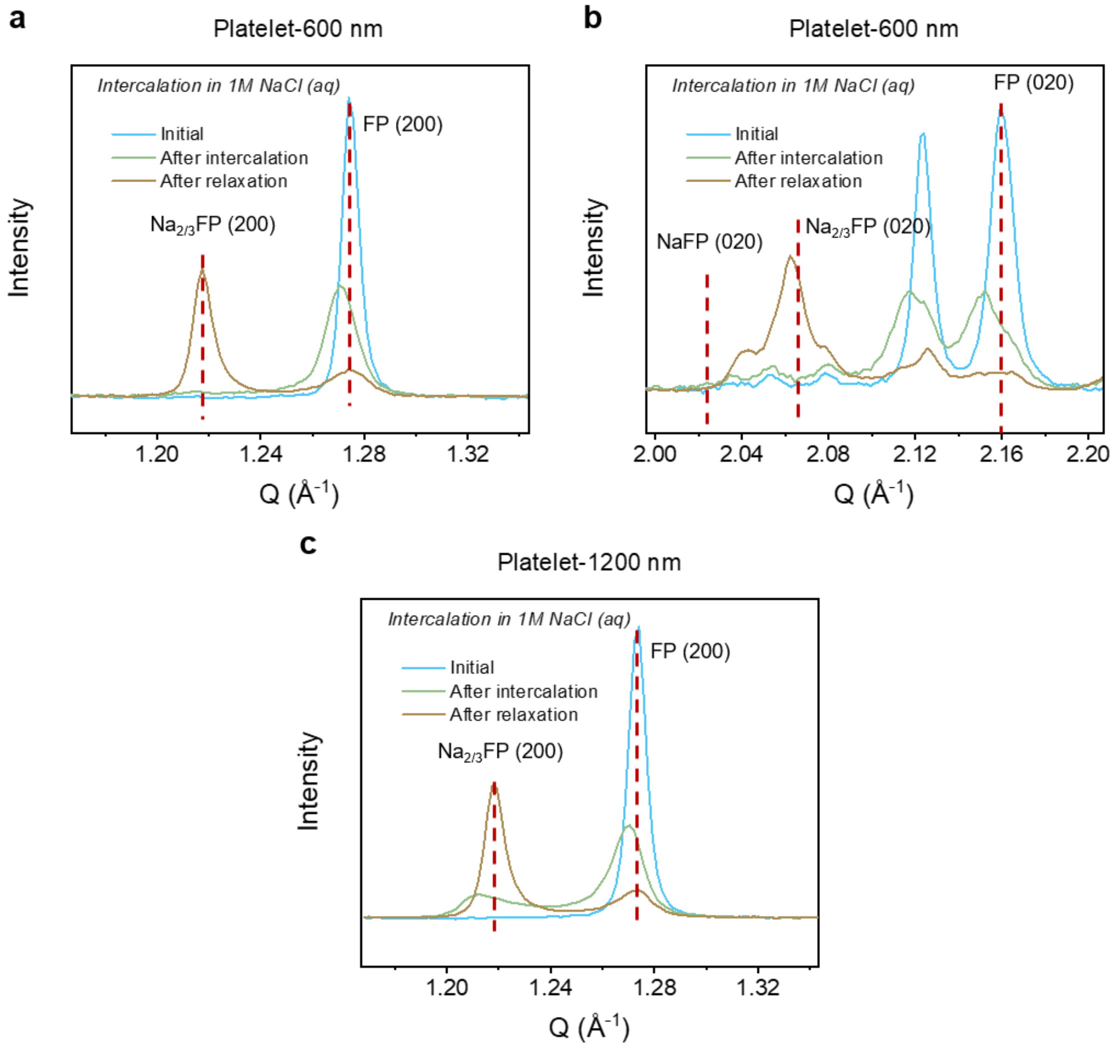

Supplementary Figure 17 Snapshots (initial scan and last scan) of *in situ* synchrotron XRD during sodiation as well as the ex-situ synchrotron XRD of the electrodes after ~ 10 hours relaxation in the open air. (a) and (b) Platelet-600 nm particles; (c) Platelet-1200 nm particles.

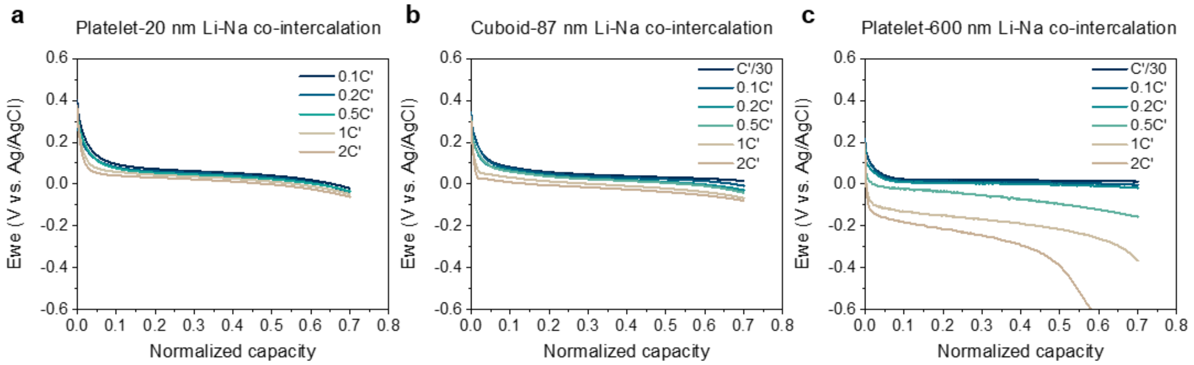

Supplementary Figure 18 Example intercalation curves of (a) Platelet-20 nm, (b) Cuboid-87 nm and (c) Platelet-600 nm particles with the use of 70% accessible capacity under different extraction rates (mass loading ~ 2.5 mg/cm^2); noting that for Platelet-600 nm at 2C' co-intercalation, only ~57% accessible capacity was used due to the reach of cut-off voltage. All the working electrodes, paired with Na_yFePO_4 counter electrodes and Ag|AgCl|KCl (4.0 M) reference electrodes, would undergo intercalation in a three-neck round-bottomed flask containing 250 mL synthetic brine solutions (1 mM LiCl and 1 M NaCl mixed solution) at room temperature (20 ~ 25 °C). N_2 (purity > 99.998%) was continuously bubbled into the solution to avoid side reactions caused from dissolved O_2.

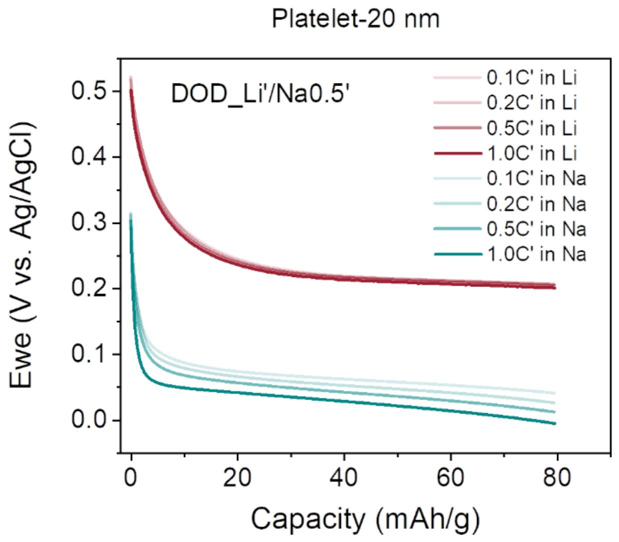

Supplementary Figure 19 Lithiation and sodiation chronopotentiometry curves of preconditioned Platelet-20 nm $FePO_4$ particles in either 60 mL 1 M LiCl aqueous solutions (paired with Li_xFePO_4 counter electrodes) or 60 mL 1 M NaCl aqueous solutions (paired with Na_yFePO_4 counter electrodes) at 0.1C'/0.2C'/0.5C'/1.0C' (0.1C' for the Platelet-20 nm particle corresponds to 15.9 mA/g) until 50% use of accessible capacity. N_2 (purity > 99.998%) was continuously bubbled into the solution to avoid side reactions caused from dissolved O_2.

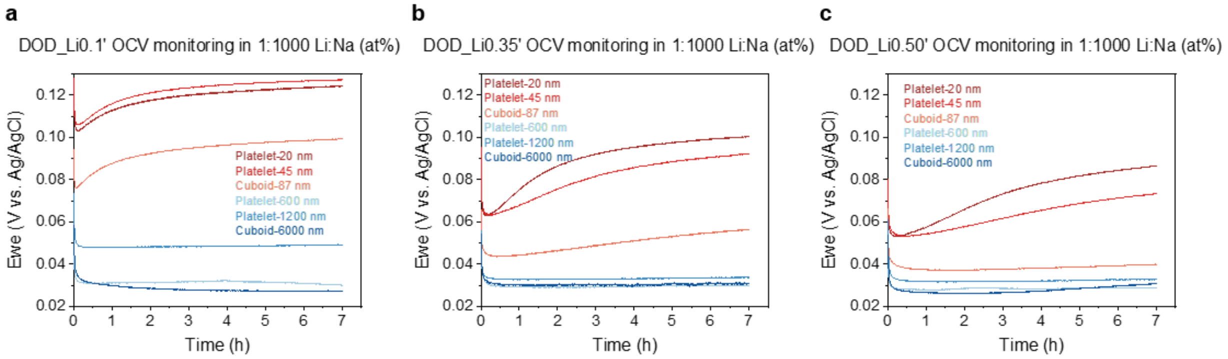

Supplementary Figure 20 Open circuit voltage (OCV) of the electrodes monitored in 1 mM LiCl and 1 M NaCl mixed solution for 7 hours, using Li pre-intercalated particles. Starting with (a) DOD_Li0.1', (b) DOD_Li0.35', or (c) DOD_Li0.5'. All the working electrodes are paired with carbon rod counter electrodes and Ag|AgCl|KCl (4.0 M) reference electrodes. N_2 (purity > 99.998%) was continuously bubbled into the solution to avoid side reactions caused from dissolved O_2.

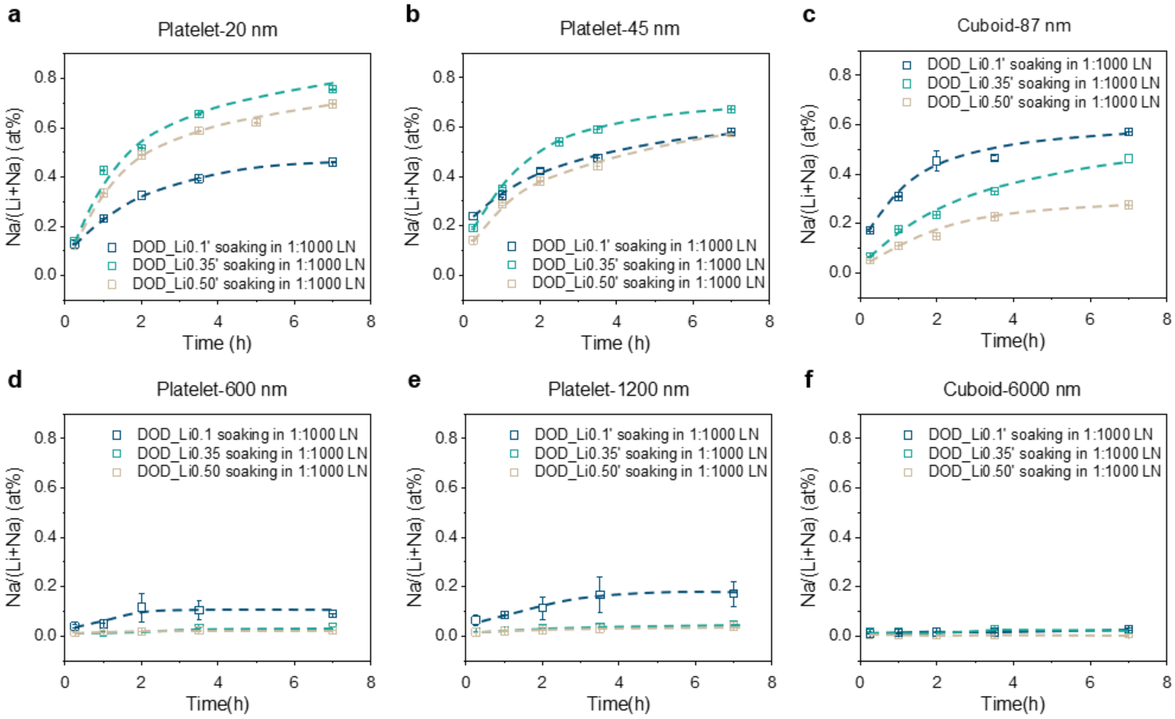

Supplementary Figure 21 Compositions evolutions with time. Measured Na/(Na+Li) ratios of (a) Platelet-20 nm, (b) Platelet-45 nm, (c) Cuboid-87 nm, (d) Platelet-600 nm, (e) Platelet-1200 nm, and (f) Cuboid-6000 nm particles after soaking in 1 mM LiCl and 1 M NaCl mixed solution for different periods, using DOD_Li0.1'/0.35'/0.5' pre-intercalated particles; dashed lines are to guide the eye.

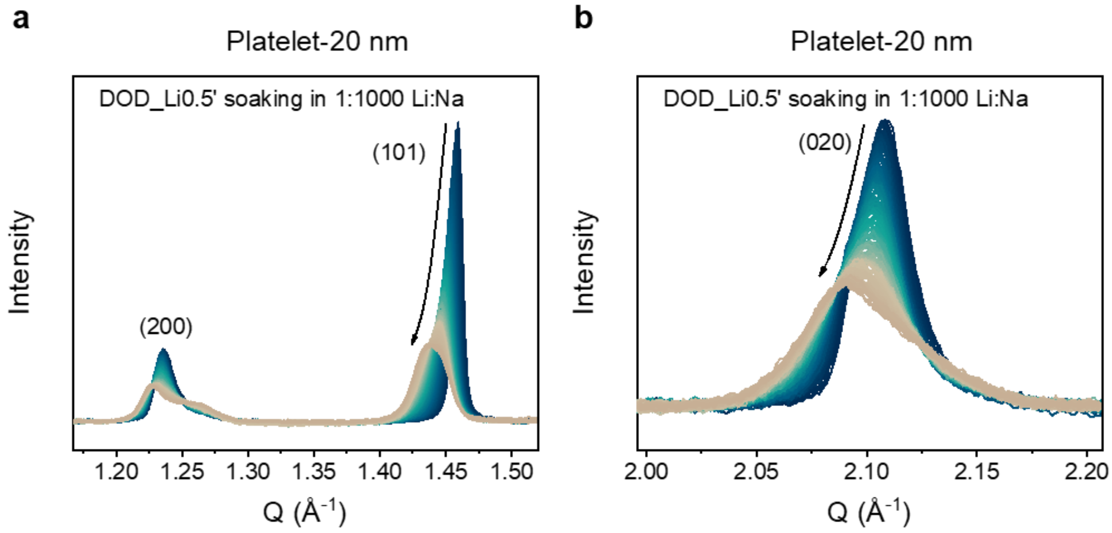

Supplementary Figure 22 *In situ* synchrotron XRD tracking of Platelet-20 nm pre-intercalated particles (DOD_Li0.50') during ion-exchange in 1 mM LiCl and 1 M NaCl mixed solution. Each scan is around 155 seconds.

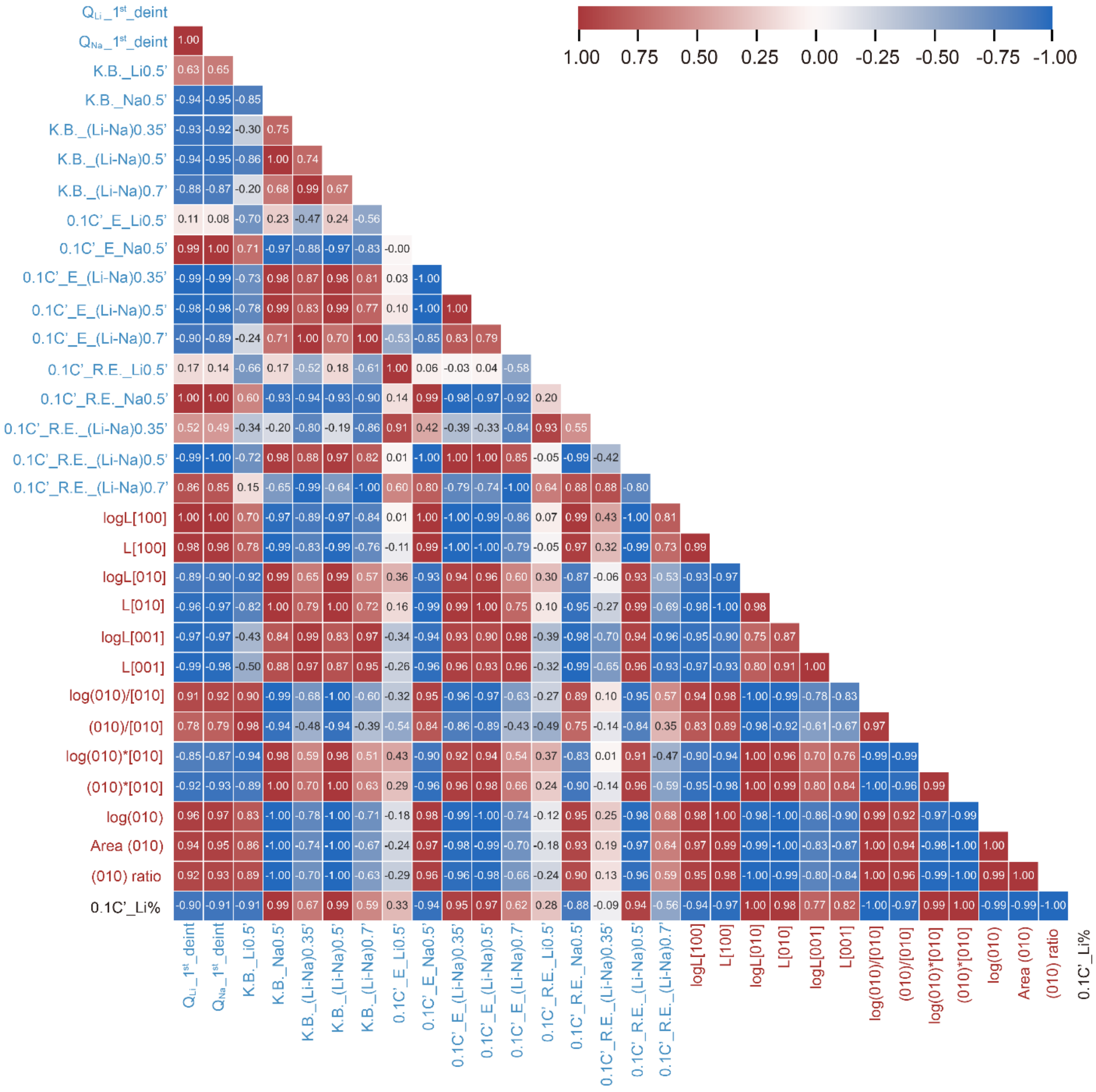

Supplementary Figure 23 Complete coefficient of correlation (R) map for particles in Group 1.

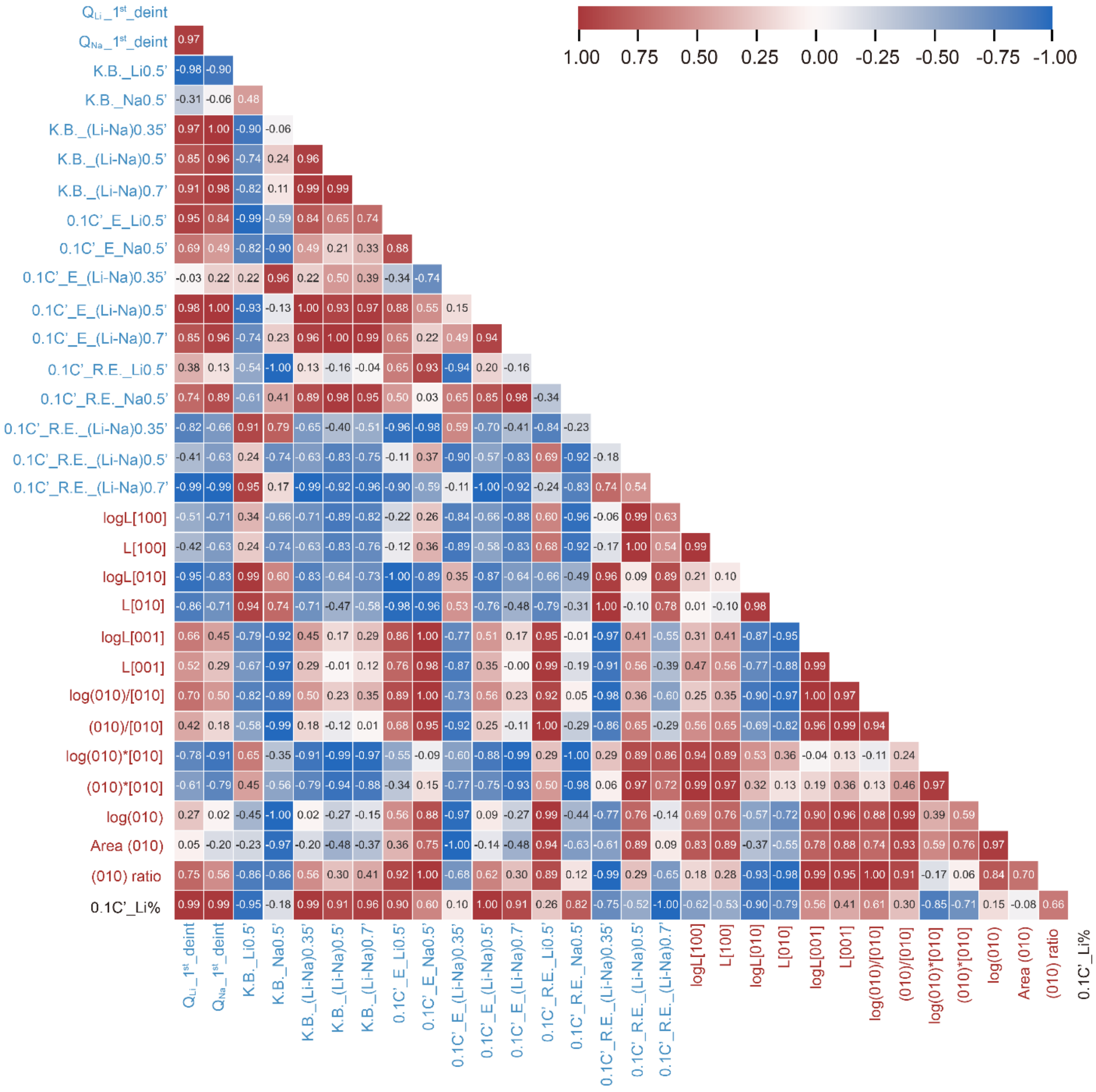

Supplementary Figure 24 Complete coefficient of correlation (R) map for particles in Group 2.

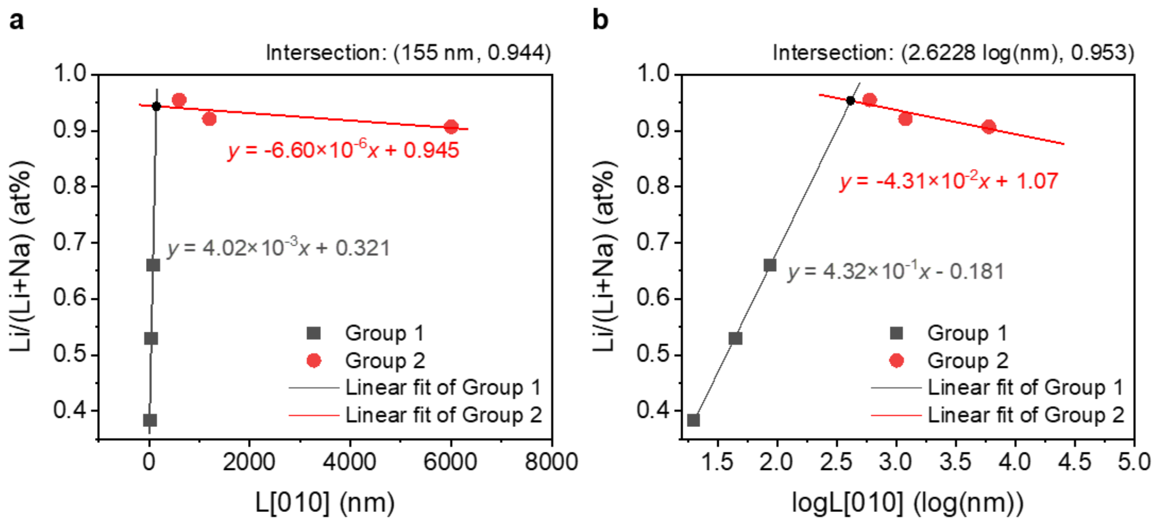

Supplementary Figure 25 Exploration of optimal channel length. (a) Recovered Li atomic ratio under 0.1C' vs. [010] channel length with the corresponding linear fit and expression of relation. (b) Recovered Li atomic ratio under 0.1C' vs. logarithmic [010] channel length with the corresponding linear fit and expression of relation. The intersection points are highlighted by black solid dots.

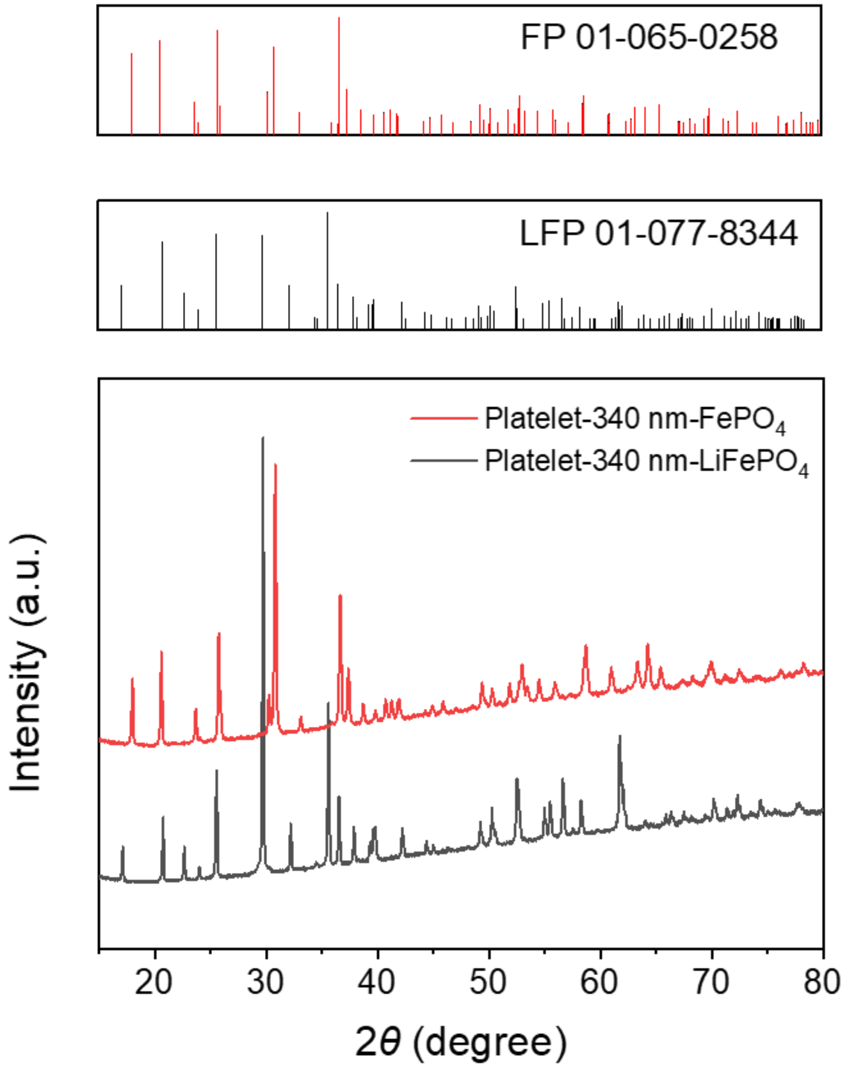

Supplementary Figure 26 XRD patterns of Platelet-340nm-$LiFePO_4$ particles and chemically extracted Platelet-340nm-$FePO_4$ particles with corresponding standard PDF cards (Olivine $LiFePO_4$ phase: 01-077-8344; Olivine $FePO_4$ phase: 01-065-0258).

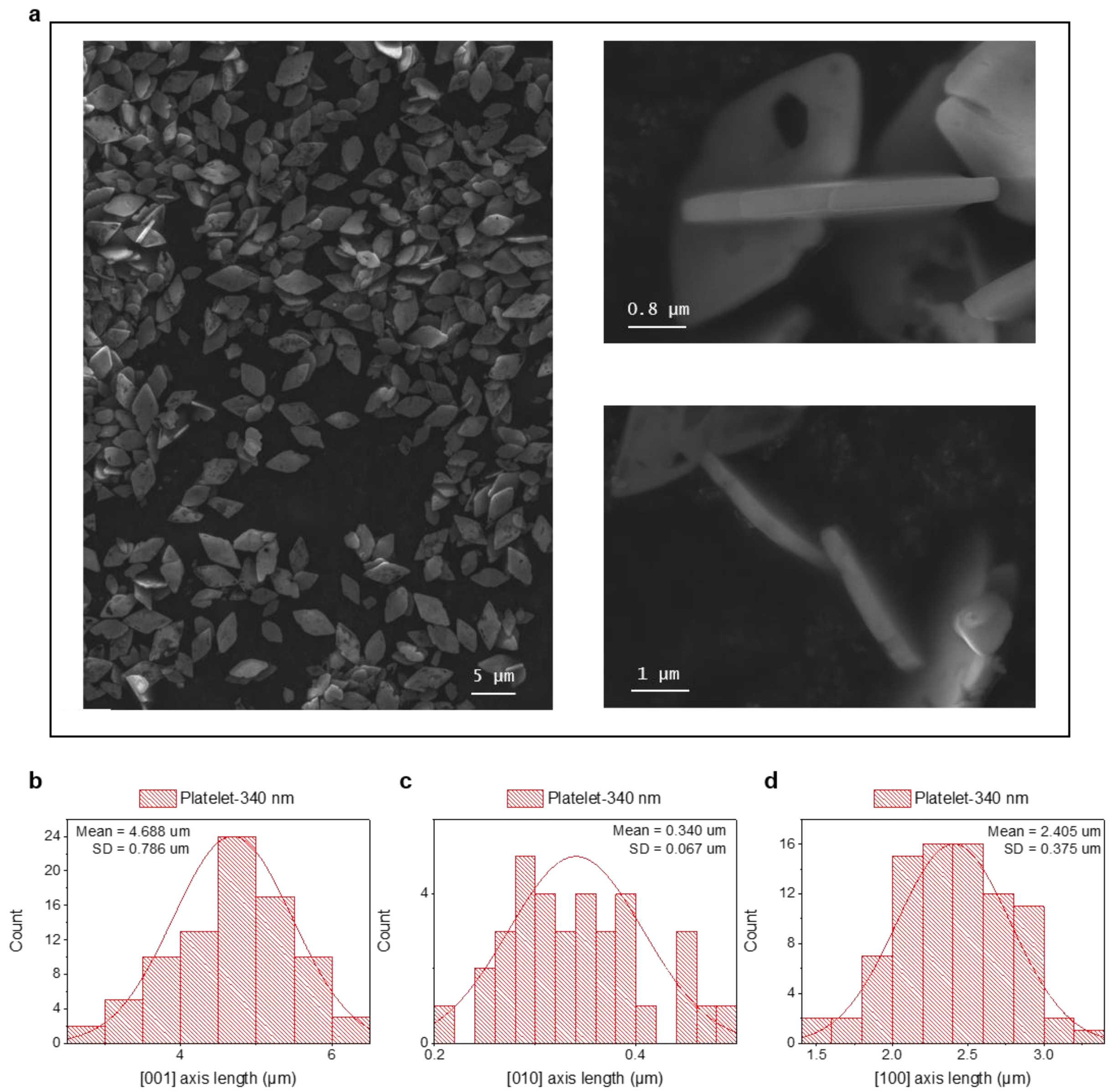

Supplementary Figure 27 Synthesized Platelet-340 nm $LiFePO_4$ particles and summarized particle dimensions. (a) SEM images. (b) Particle dimension distribution along the [001] axis. (c) Particle dimension distribution along the [010] axis. (d) Particle dimension distribution along the [100] axis. (Only particles with fully exposed dimensions were counted.)

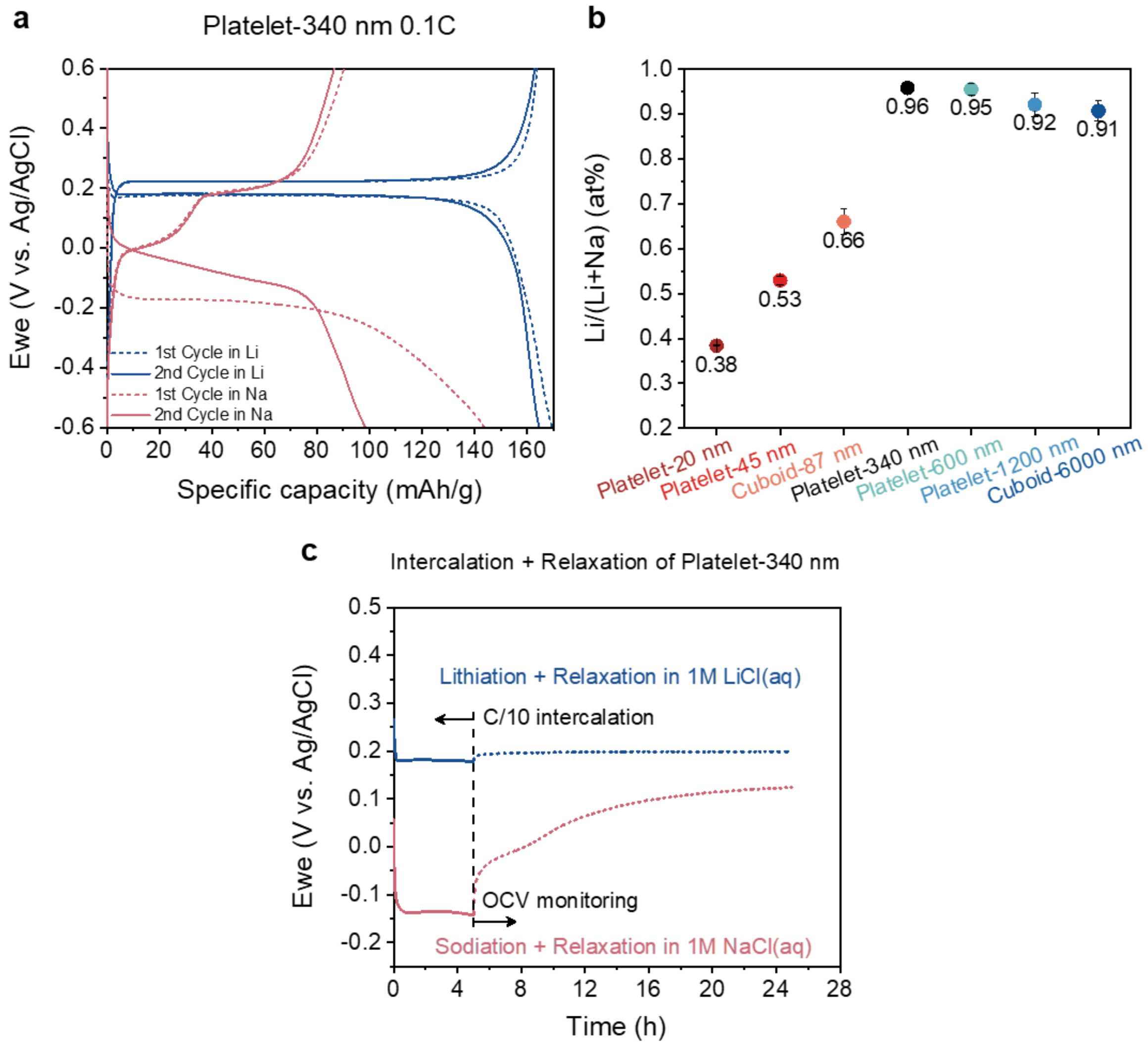

Supplementary Figure 28 Electrochemical response and Li extraction performance of Platelet-340 nm particles. (a) Electrochemical cycling of the chemically extracted Platelet-340 nm-$FePO_4$ electrodes in either 60 mL 1 M LiCl aqueous solutions (17 mA/g; paired with Li_xFePO_4 counter electrodes) or 60 mL 1 M NaCl aqueous solutions (15.4 mA/g; paired with Na_yFePO_4 counter electrodes) between - 0.6 V and 0.6 V (vs. Ag/AgCl/KCl (4.0 M)) at room temperature (20 ~ 25 °C). N_2 (purity > 99.998%) was continuously bubbled into the solution to avoid side reactions caused from dissolved O_2. (b) Li/(Li+Na) ratios after recovery of different electrodes from 1:1000 Li to Na solution using 70% accessible capacity and 0.1C' extraction rate. Error bars represent the standard deviation of three replicate measurements. (c) 0.1C' lithiation or sodiation until 50% accessible capacity then 20hrs OCV monitoring.

Supplementary Table 1 Calculated surface energies of $LiFePO_4$ in seven low-index orientations together with values for (201) and (301) surfaces.

Orientation	(100)	(010)	(001)	(101)	(011)	(110)	(111)	(201)	(301)
γ (J/m^2)	0.466	0.569	1.019	0.868	0.828	1.063	0.990	0.459	0.670

Supplementary Table 2 Fitted parameters from diffraction patterns in Supplementary Figure 7.

	Platelet-20 nm-$LiFePO_4$	Platelet-45 nm-$LiFePO_4$	Cuboid-87 nm-$LiFePO_4$	Platelet-600 nm-$LiFePO_4$	Platelet-1200 nm-$LiFePO_4$	Cuboid-6000 nm-$LiFePO_4$
a (Å)	10.302252	10.305038	10.314738	10.323062	10.327748	10.331326
b (Å)	5.998117	5.995944	6.000506	6.004919	6.008061	6.008345
c (Å)	4.693041	4.694987	4.695092	4.691414	4.695745	4.694746
α	90					
β						
γ						

Supplementary Table 3 Summarized morphology features from Supplementary Figures 1-6. See Supplementary Note 3 for the calculations of (010)/[010] and (010) ratio. (Only average values are summarized in the table.)

Shape features	L[100] (nm)	L[010] (nm)	L[001] (nm)	(010)/[010] (nm)	(010)*[010] (nm^3)	(010) ratio
Platelet-20 nm	83.0	20.0	83.0	628	2.51E+05	0.70
Platelet-45 nm	64.0	45.0	64.0	237	4.80E+05	0.46
Cuboid-87 nm	24.0	87.0	377	95.0	7.19E+05	0.21
Platelet-600 nm	1.36E+03	600	6.41E+03	8.79E+03	3.16E+09	0.57
Platelet-1200 nm	3.01E+03	1.20E+03	9.27E+03	1.67E+04	2.40E+10	0.62
Cuboid-6000 nm	1.90E+03	6.00E+03	1.90E+03	391	1.41E+10	0.12

Supplementary Table 4 Fitted parameters from diffraction patterns in Supplementary Figure 8.

	Platelet-20 nm-$FePO_4$	Platelet-45 nm-$FePO_4$	Cuboid-87 nm-$FePO_4$	Platelet-600 nm-$FePO_4$	Platelet-1200 nm-$FePO_4$	Cuboid-6000 nm-$FePO_4$
a (Å)	9.816781	9.815551	9.819198	9.820441	9.816989	9.820099
b (Å)	5.810195	5.809385	5.807198	5.794683	5.796631	5.788165
c (Å)	4.784253	4.783833	4.780628	4.780468	4.780244	4.777556
α	90					
β						
γ						

Supplementary Table 5 Delivered capacity in the first charge and discharge during 0.1C lithiation (17 mA/g) and sodiation (15.4 mA/g), summarized from Figures 3a and 3b. The delivered capacity in the first charge process is also identified as the accessible capacity. For some experiments, the calculations of applied current and depth of discharge are both based on the delivered capacity in the first de-lithiation instead of the theoretical capacity. To differentiate the C-rates calculated using different approaches, we denote the C-rates as C' instead of C.

	Q_{Li}_1st_int (mAh/g)	Q_{Li}_1st_deint (mAh/g)	Q_{Na}_1st_int (mAh/g)	Q_{Na}_1st_deint (mAh/g)	Accessible capacity (mAh/g)	0.1C' (mA/g)
Platelet-20 nm	162	159	144	119	159	15.9
Platelet-45 nm	161	158	139	116	158	15.8
Cuboid-87 nm	158	153	150	95	153	15.3
Platelet-600 nm	162	159	150	88	159	15.9
Platelet-1200 nm	156	141	115	74	141	14.1
Cuboid-6000 nm	153	129	130	71	129	12.9

Supplementary Table 6 Summarized potential difference at the halfway capacity point of the initial discharge under 0.1C (17 mA/g for lithiation and 15.4 mA/g for sodiation; labeled as 0.1C-E_Li or labeled as 0.1C-E_Na) from Figures 3a and 3b. The potential difference between lithiation and sodiation is also calculated in the table.

	0.1C-E_Li (V vs. Ag/AgCl)	0.1C-E_Na (V vs. Ag/AgCl)	0.1C-E_Li-Na (V vs. Ag/AgCl)
Platelet-20 nm	0.204	0.0173	0.187
Platelet-45 nm	0.205	0.00911	0.196
Cuboid-87 nm	0.204	-0.0114	0.215
Platelet-600 nm	0.193	-0.323	0.516
Platelet-1200 nm	0.196	-0.123	0.319
Cuboid-6000 nm	0.149	-0.341	0.490

Supplementary Table 7 Summarized end intercalation potential and potential change/kinetic barrier from Supplementary Figure 11 during constant current lithiation or sodiation and the following 20 hours relaxation. The potential change difference between lithiation and sodiation is also calculated in the table.

	0.1C'_E _Li0.35'	0.1C'_E _Li0.5'	0.1C'_E _Li0.7'	0.1C'_E _Na0.35'	0.1C'_E _Na0.5'	0.1C'_E _Na0.7'	0.1C'_E _(Li-Na)0.35'	0.1C'_E _(Li-Na)0.5'	0.1C'_E _(Li-Na)0.7'
	End intercalation potential at certain DOD (V vs. Ag/AgCl)						End intercalation potential difference at the same DOD (V)		
Platelet-20 nm	0.215	0.206	0.198	0.0640	0.0405	-0.0251	0.151	0.166	0.223
Platelet-45 nm	0.216	0.211	0.199	0.0554	0.0309	-0.0169	0.161	0.180	0.216
Cuboid-87 nm	0.211	0.208	0.200	0.0223	-0.00290	-0.0382	0.189	0.211	0.238
Platelet-600 nm	0.196	0.193	0.188	-0.108	-0.128	-0.161	0.304	0.321	0.349
Platelet-1200 nm	0.192	0.171	0.0662	-0.0900	-0.121	-0.193	0.282	0.293	0.260
Cuboid-6000 nm	0.149	0.124	0.0841	-0.158	-0.159	-0.188	0.307	0.284	0.272
	K.B._Li0.35'	K.B._Li0.5'	K.B._Li0.7'	K.B._Na0.35'	K.B._Na0.5'	K.B._Na0.7'	K.B._(Li-Na)0.35'	K.B._(Li-Na)0.5'	K.B._(Li-Na)0.7'
	Kinetic barrier measured/Potential change during relaxation (V)						Potential change difference at the same DOD (V)		
Platelet-20 nm	0.00273	0.00555	0.00482	0.0766	0.107	0.0911	0.0739	0.101	0.0863
Platelet-45 nm	0.00406	0.00525	0.00634	0.0615	0.114	0.0760	0.0575	0.109	0.0697
Cuboid-87 nm	0.00441	0.00523	0.00794	0.120	0.125	0.122	0.115	0.119	0.114
Platelet-600 nm	0.00784	0.0107	0.0157	0.166	0.207	0.239	0.158	0.196	0.224
Platelet-1200 nm	0.0194	0.0381	0.143	0.155	0.181	0.248	0.136	0.143	0.105
Cuboid-6000 nm	0.0487	0.0744	0.116	0.180	0.225	0.222	0.131	0.151	0.107

Supplementary Table 8 Particle features and the corresponding definition.

Feature	Label	Definition
Morphology	L[100]	Length along the [100] axis
	L[010]	Length along the [010] axis
	L[001]	Length along the [001] axis
	(010)/[010]	(010) facet area over [010] channel length ratio
	(010)*[010]	Particle volume
	Area (010)	(010) facet exposure area
	(010) ratio	(010) facet exposure area over total surface area
	logL[100]	Logarithmic length along the [100] axis
	logL[010]	Logarithmic length along the [010] axis
	logL[001]	Logarithmic length along the [001] axis
	log(010)/[010]	Logarithmic ratio of (010) facet area over [010] channel length
	log(010)*[010]	Logarithmic particle volume
	Log(010)	Logarithmic (010) facet exposure area
Property	Q_{Li}_1^{st}_deint	Delivered capacity in the first 0.1C (17 mA/g) de-lithiation process
	Q_{Na}_1^{st}_deint	Delivered capacity in the first 0.1C (15.4 mA/g) de-sodiation process
	K.B._Li0.5’	Measured kinetic barrier/potential change during 20 hours relaxation after 0.1C’ constant current lithiation to Li0.5’
	K.B._Na0.5’	Measured kinetic barrier/potential change during 20 hours relaxation after 0.1C’ constant current sodiation to Na0.5’
	K.B._(Li-Na)0.35’	Difference between K.B._Li0.35’ and K.B._Na0.35’
	K.B._(Li-Na)0.5’	Difference between K.B._Li0.5’ and K.B._Na0.5’

	K.B._(Li-Na)0.7’	Difference between K.B._Li0.5’ and K.B._Na0.7’
	0.1C’_E_Li0.5’	End potential after 0.1C’ constant current lithiation to Li0.5’
	0.1C’_E_Na0.5’	End potential after 0.1C’ constant current sodiation to Na0.5’
	0.1C’_E_(Li-Na)0.35’	Difference between 0.1C’_E_Li0.35’ and 0.1C’_E_Na0.35’
	0.1C’_E_(Li-Na)0.5’	Difference between 0.1C’_E_Li0.5’ and 0.1C’_E_Na0.5’
	0.1C’_E_(Li-Na)0.7’	Difference between 0.1C’_E_Li0.7’ and 0.1C’_E_Na0.7’
	0.1C’_R.E._Li0.5’	End potential after 20 hours relaxation of Li0.5’
	0.1C’_R.E._Na0.5’	End potential after 20 hours relaxation of Na0.5’
	0.1C’_R.E._(Li-Na)0.35’	Difference between 0.1C’_R.E._Li0.35’ and 0.1C’_R.E._Na0.35’
	0.1C’_R.E._(Li-Na)0.5’	Difference between 0.1C’_R.E._Li0.5’ and 0.1C’_R.E._Na0.5’
	0.1C’_R.E._(Li-Na)0.7’	Difference between 0.1C’_R.E._Li0.7’ and 0.1C’_R.E._Na0.7’
**Performance**	0.1C’_Li%	Recovered Li/(Li+Na) atomic ratios using 70% accessible capacity, 0.1C' extraction rate, and ~2.5 mg/cm^2 mass loading

Supplementary Table 9 Summarized features of the six particles.

Feature		Platelet-20 nm	Platelet-45 nm	Cuboid-87 nm	Platelet-600 nm	Platelet-1200 nm	Cuboid-6000 nm
Morphology	L[100] (nm)	83.0	64.0	24.0	1.36E+03	3.01E+03	1.90E+03
	L[010] (nm)	20.0	45.0	87.0	600	1.20E+03	6.00E+03
	L[001] (nm)	83.0	64.0	377	6.41E+03	9.27E+03	1.90E+03
	(010)/[010] (nm)	628	237	95.0	8.79E+03	1.67E+04	391
	(010)*[010] (nm^3)	2.51E+05	4.80E+05	7.19E+05	3.16E+09	2.40E+10	1.41E+10
	Area (010) (nm^2)	1.26E+04	1.07E+04	8.26E+03	5.27E+06	2.00E+07	2.35E+06
	(010) ratio	0.70	0.46	0.21	0.57	0.62	0.12
	logL[100] (log(nm))	1.92	1.81	1.38	3.13	3.48	3.28
	logL[010] (log(nm))	1.30	1.65	1.94	2.78	3.08	3.78
	logL[001] (log(nm))	1.92	1.81	2.58	3.81	3.97	3.28
	log(010)/[010] (log(nm))	2.80	2.38	1.98	3.94	4.22	2.59
	log(010)*[010] (log(nm^3))	5.40	5.68	5.86	9.50	10.4	10.1
	Log(010) (log(nm^2))	4.10	4.03	3.92	6.72	7.30	6.37
Property	Q_{Li}_1st_deint (mAh/g)	159	158	153	159	141	129
	Q_{Na}_1st_deint (mAh/g)	119	116	94.9	88.4	73.7	70.5

	K.B._Li0.5’ (V)	0.00560	0.00530	0.00520	0.0107	0.0381	0.0744
	K.B._Na0.5’ (V)	0.107	0.114	0.125	0.207	0.181	0.225
	K.B._(Li-Na)0.35’ (V)	0.0739	0.0575	0.115	0.158	0.136	0.131
	K.B._(Li-Na)0.5’ (V)	0.101	0.109	0.119	0.196	0.143	0.151
	K.B._(Li-Na)0.7’ (V)	0.0863	0.0697	0.114	0.224	0.105	0.107
	0.1C’_E_Li0.5’ (V)	0.206	0.211	0.208	0.193	0.171	0.124
	0.1C’_E_Na0.5’ (V)	0.0405	0.0309	-0.00300	-0.128	-0.121	-0.159
	0.1C’_E_(Li-Na)0.35’ (V)	0.152	0.161	0.189	0.304	0.282	0.307
	0.1C’_E_(Li-Na)0.5’ (V)	0.166	0.180	0.211	0.322	0.293	0.284
	0.1C’_E_(Li-Na)0.7’ (V)	0.223	0.216	0.238	0.349	0.260	0.272
	0.1C’_R.E._Li0.5’ (V)	0.212	0.216	0.213	0.204	0.210	0.199
	0.1C’_R.E._Na0.5’ (V)	0.147	0.145	0.122	0.0788	0.0598	0.0658
	0.1C’_R.E._(Li-Na)0.35’ (V)	0.0776	0.103	0.0736	0.146	0.147	0.176
	0.1C’_R.E._(Li-Na)0.5’ (V)	0.0649	0.0709	0.0914	0.125	0.150	0.133
	0.1C’_R.E._(Li-Na)0.7’ (V)	0.137	0.146	0.124	0.126	0.154	0.166
**Performance**	0.1C’_Li%	0.384	0.529	0.660	0.955	0.921	0.907

Supplementary Table 10 Summarized features of the Platelet-340 nm particles.

	L[010] (nm)	logL[010] (log(nm))	K.B._(Li-Na)0.5' (V)	0.1C'_E_(Li-Na)0.5' (V)	0.1C'-R.E._(Li-Na)0.5' (V)	0.1C'_Li%
Platelet-20 nm	20.0	1.30	0.101	0.166	0.0649	0.384
Platelet-45 nm	45.0	1.65	0.109	0.180	0.0709	0.529
Cuboid-87 nm	87.0	1.94	0.119	0.211	0.0914	0.660
Platelet-340 nm	200	2.30	0.245	0.321	0.284	0.959
Platelet-600 nm	600	2.78	0.196	0.322	0.125	0.955
Platelet-1200 nm	1.20E+3	3.08	0.143	0.293	0.150	0.921
Cuboid-6000 nm	6.00E+3	3.79	0.151	0.284	0.133	0.907

Supplementary Note 1: Calculations for surface energy, bulk/surface redox potential, and construction of Wulff shape

First-principles density functional theory (DFT) calculations were performed using the Vienna ab initio simulation package (VASP) employing the projector augmented-wave (PAW) method[1-3]. The Perdew-Burke-Ernzerhof (PBE) generalized-gradient approximation (GGA) was employed to describe the exchange-correlation energy[4,5]. A plane-wave cut-off energy of 520 eV was utilized, along with a Gamma-centered k-point mesh, ensuring at least 1500 k-point density per reciprocal atom was used. Spin-polarized calculations were initiated with a high-spin configuration for all relaxations[6]. The Hubbard U parameter was applied to correct the on-site Coulomb interactions for the localized Fe 3d orbitals[7]. The U values for Fe were set at 5.3 eV, determined from the Materials Project by fitting the formation enthalpies of the binary transition metal oxides to their experimental values[8,9]. We did not vary the U values of Fe for different Li or Na – vacancy ratios since this effect is negligible and consistent with our previous DFT calculations[10,11]. The total energy was converged to 10^{-5} eV per supercell, and the forces were kept below 0.02 eV/ Å.

The surface energy and surface redox potential we calculated for the $LiFePO_4$ (LFP), $NaFePO_4$ (NFP), and $Na_{0.67}FePO_4$ ($N_{0.67}FP$) generally follow the same procedure as previous DFT studies[10,12]. For the surface calculations, slab model that ensure at least four surface layers and 15 Å vacuum layer thickness are consistently used. The innermost layer of the surface layers is kept frozen at the bulk position to simulate the bulk of the corresponding surface. Symmetrically equivalent operation is applied to the slab model by an inversion, mirror, or glide operation to avoid dipole moment that can result in significant surface energy[13]. Furthermore, the PO_4 tetrahedron is always protected from being cut through at the surface termination layer. The equation used to calculate surface energy (γ) and surface redox potential (V) (take LFP as an example) is as follows:

$$\gamma = \frac{E_{slab} - nE_{bulk}}{2A} \quad (1)$$

where E_{slab} and E_{bulk} are the DFT calculated total energy of the slab and the corresponding bulk. n is the number of formula units, and A is the surface area of the surface.

$$V = -\frac{E_{LFP} - E_{FP}}{n_{Li}} + \mu_{Li} \quad (2)$$

where E_{LFP} and E_{FP} are the DFT calculated total energy of LFP and $FePO_4$ (FP) in the slab model. n_{Li} is the number of formula units, and μ_{Li} is the chemical potential of *bcc* Li that serves as the anode reference.

The Wulff construction provides insights into the equilibrium shape of a crystal by examining its surface energies[14]. A series of planes are generated by drawing a plane perpendicular to each vector and passing it through the endpoint. The Wulff construction is formed by the inner envelope of these planes[15].

Supplementary Note 2: Solvothermal synthesis of $LiFePO_4$ particles

Based on other reports, a solvothermal synthesis method was used to synthesize all six pristine $LiFePO_4$ particles, each with a slightly different recipe[16-18]. Unless specified, all the mixing and dissolving operations were done in an N_2/H_2O glovebox (< 1 ppm O_2) to ensure that all precursors were not exposed to oxygen.

For Platelet-20 nm particles:
To ensure the reactants were well dissolved, the ethylene glycol (EG) used in the experiment was pre-heated to 60°C. 6 mmol H_3PO_4 and 16.2 mmol $LiOH·H_2O$ were first dissolved in 20 ml and 15 ml EG, respectively, stirring overnight. Meanwhile, 6 mmol of $FeSO_4·7H_2O$ was dried overnight at 45°C to deoxygenate. Afterward, the dried $FeSO_4$ powder was slowly added to the H_3PO_4-EG mixture with continuous stirring to ensure complete dissolution. Next, the LiOH-EG solution was slowly added into the $FeSO_4$-H_3PO_4-EG solution with constant stirring for five minutes. At last, the mixture was performed into a 100 ml autoclave without oxygen exposure and heated at 180°C (ramp rate 3°C/min) for 10 hours. The Platelet-20 nm $LiFePO_4$ particles were formed in the bottom.

For Platelet-45 nm particles:
To ensure the reactants were well dissolved, the EG used in the experiment was pre-heated to 60°C. 6 mmol H_3PO_4 and 15 mmol $LiOH·H_2O$ were first dissolved in 20 ml and 15 ml EG, respectively, stirring overnight. Meanwhile, 6 mmol of $FeSO_4·7H_2O$ was dried overnight at 45°C to deoxygenate. Afterward, the dried $FeSO_4$ powder was slowly added to the H_3PO_4-EG mixture with continuous stirring to ensure complete dissolution. Next, the LiOH-EG solution was slowly added into the $FeSO_4$-H_3PO_4-EG solution with constant stirring for five minutes. At last, the mixture was performed into a 100 ml autoclave without oxygen exposure and heated at 180°C (ramp rate 3°C/min) for 10 hours. The Platelet-45 nm $LiFePO_4$ particles were formed in the bottom.

For Cuboid-87 nm particles:
To ensure the reactants were well dissolved, the EG used in the experiment was pre-heated to 60°C. 6 mmol H_3PO_4 and 18 mmol $LiOH·H_2O$ were first dissolved in 10 ml and 15 ml EG, respectively, stirring overnight. Meanwhile, 6 mmol of $FeSO_4·7H_2O$ was dried overnight at 45°C to deoxygenate. Afterward, the dried $FeSO_4$ powder was dissolved in 10 ml EG with continuous stirring to ensure complete dissolution. And LiOH-EG solution was slowly added into the H_3PO_4-EG solution to produce the creamy-white Li_3PO_4 suspension. Next, the $FeSO_4$-EG solution was transferred to the Li_3PO_4 suspension with constant stirring for five minutes. At last, the mixture was performed into a 100 ml autoclave without oxygen exposure and heated at 180°C (ramp rate 3°C/min) for 10 hours. The Cuboid-87 nm $LiFePO_4$ particles were formed in the bottom.

For Platelet-600 nm particles:
To ensure the reactants were well dissolved, the polyethylene glycol 400 (PEG400) used in the experiment

was pre-heated to 60℃. 1.2 mmol H_3PO_4 and 3.6 mmol $LiOH\cdot H_2O$ were first dissolved in 24 ml and 18 ml PEG400, respectively. Afterward, the LiOH-PEG400 mixture was added to the H_3PO_4-PEG400 solution to produce the creamy-white Li_3PO_4 precipitate. This mixture was stirred overnight in the glovebox to remove dissolved oxygen. Meanwhile, 1.2 mmol of $FeSO_4\cdot 7H_2O$ was dried overnight at 45℃ to deoxygenate, followed by dissolving in 12 ml deoxygenated PEG400. The $FeSO_4$ mixture was transferred to the Li_3PO_4 suspension, and the entire mixture was transferred to a 100 mL Teflon-lined autoclave without oxygen exposure. The autoclave was initially heated to 125℃ for 1 h, then to 210℃ for 17 h, and cooled. The Platelet-600 nm $LiFePO_4$ particles were formed in the bottom.

For Platelet-1200 nm particles:
To ensure the reactants were well dissolved, the H_2O-PEG400 co-solvent (in a volumetric ratio of 1:2) used in the experiment was pre-heated to 60℃. 6 mmol H_3PO_4 and 16.2 mmol $LiOH\cdot H_2O$ were first dissolved in 20 ml and 15 ml H_2O-PEG400 co-solvent, respectively, stirring overnight. Meanwhile, 6 mmol of $FeSO_4\cdot 7H_2O$ was dried overnight at 45℃ to deoxygenate. Afterward, the dried $FeSO_4$ powder was slowly added to the H_3PO_4-H_2O-PEG400 mixture with continuous stirring to ensure complete dissolution. Next, the LiOH-H_2O-PEG400 solution was slowly added into the $FeSO_4$-H_3PO_4-H_2O-PEG400 solution with constant stirring for five minutes. At last, the mixture was performed into a 50 ml autoclave without oxygen exposure and heated at 180 ℃ (ramp rate 3 ℃/min) for 17 hours. The Platelet-1200 nm $LiFePO_4$ particles were formed in the bottom.

For Cuboid-6000 nm particles:
The synthesis of Cuboid-6000 nm particles was based on one previous report[17]. Specifically, 6 mmol of ammonium dihydrogen phosphate ($NH_4H_2PO_4$), $LiOH\cdot H_2O$, and nitrilotriacetic acid [$N(CH_2CO_2H)_3$] were weighed out and transferred into a glass jar, to which 60 ml of DI water was then added and the solution was stirred for 15 min. Next, 1-2 ml of concentrated $NH_3\cdot H_2O$ was micropipetted into the solution until a pH of 9 was attained. Then the mixture, together with 6 mmol of $FeSO_4\cdot 7H_2O$, was transferred and deoxygenated in the glovebox. Afterward, the $FeSO_4\cdot 7H_2O$ was added to the mixture and the suspension was stirred for another 15 min. At last, the suspension was performed into a 100 ml autoclave without oxygen exposure and heated at 200 ℃ (ramp rate 3 ℃/min) for 24 hours and allowed to cool down naturally. The Cuboid-6000 nm $LiFePO_4$ particles were formed in the bottom.

For Platelet-340 nm particles:
To ensure the reactants were well dissolved, the polyethylene glycol 400 (PEG400) used in the experiment was pre-heated to 60℃. 6 ml of 1 M $H_3PO_{4(aq)}$ was mixed with 24 ml PEG400. Afterwards, 18 ml of 1 M $LiOH\cdot H_2O_{(aq)}$ was added to produce the creamy-white Li_3PO_4 precipitate. This mixture was stirred overnight in the glovebox to remove dissolved oxygen. Meanwhile, 6 mmol of $FeSO_4\cdot 7H_2O$ was dried overnight at 45℃ to deoxygenate, followed by dissolving in 12 ml deoxygenated water. The $FeSO_4$ mixture

was transferred to the Li_3PO_4 suspension, and the entire mixture was transferred to a 100 mL Teflon-lined autoclave without oxygen exposure. The autoclave was initially heated to 140℃ for 1 h, then to 210℃ for 17 h, and cooled. The Platelet-340 nm $LiFePO_4$ particles were formed in the bottom.

Supplementary Note 3: Calculations of (010)/[010] and (010) ratio

Here are the calculations of the (010)/[010] and (010) ratios for each particle, noting that the length is not to scale, and the assumed values are labeled in blue.

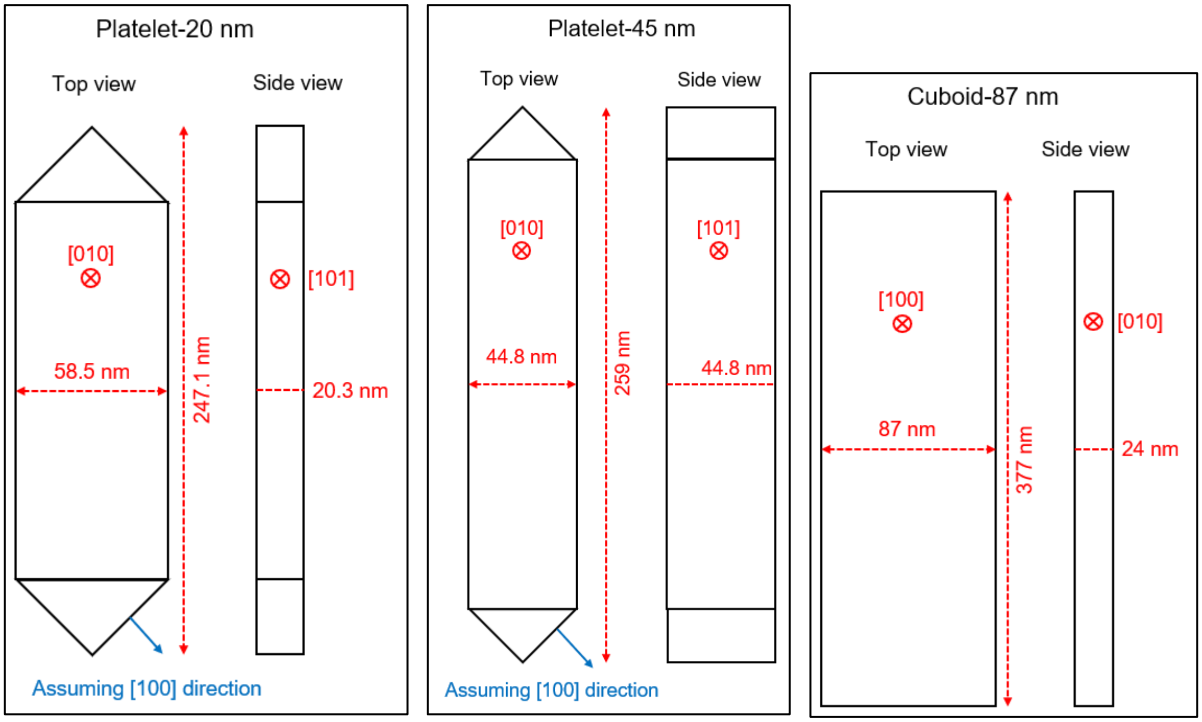

For Platelet-20 nm particles:

$$\frac{(010)}{[010]} = \frac{(247.1 - 29.25) \times 58.5}{20.3} = 627.8 \text{ nm}$$

$$(010) \text{ ratio} = \frac{(247.1 - 29.25) \times 58.5 \times 2}{[(247.1 - 29.25) \times 58.5 \times 2 + (41.366 \times 4 + 188.6 \times 2) \times 20.3]} = 0.698$$

For Platelet-45 nm particles:

$$\frac{(010)}{[010]} = \frac{(259 - 22.4) \times 44.8}{44.8} = 236.6 \text{ nm}$$

$$(010) \text{ ratio} = \frac{(259 - 22.4) \times 44.8 \times 2}{[(259 - 22.4) \times 44.8 \times 2 + (31.678 \times 4 + 214.2 \times 2) \times 44.8]} = 0.460$$

For Cuboid-87 nm particles:

$$\frac{(010)}{[010]} = \frac{377 \times 24}{87} = 94.90 \text{ nm}$$

$$(010)\ \text{ratio} = \frac{377 \times 24 \times 2}{(377 \times 24 \times 2 + 377 \times 87 \times 2 + 24 \times 87 \times 2)} = 0.206$$

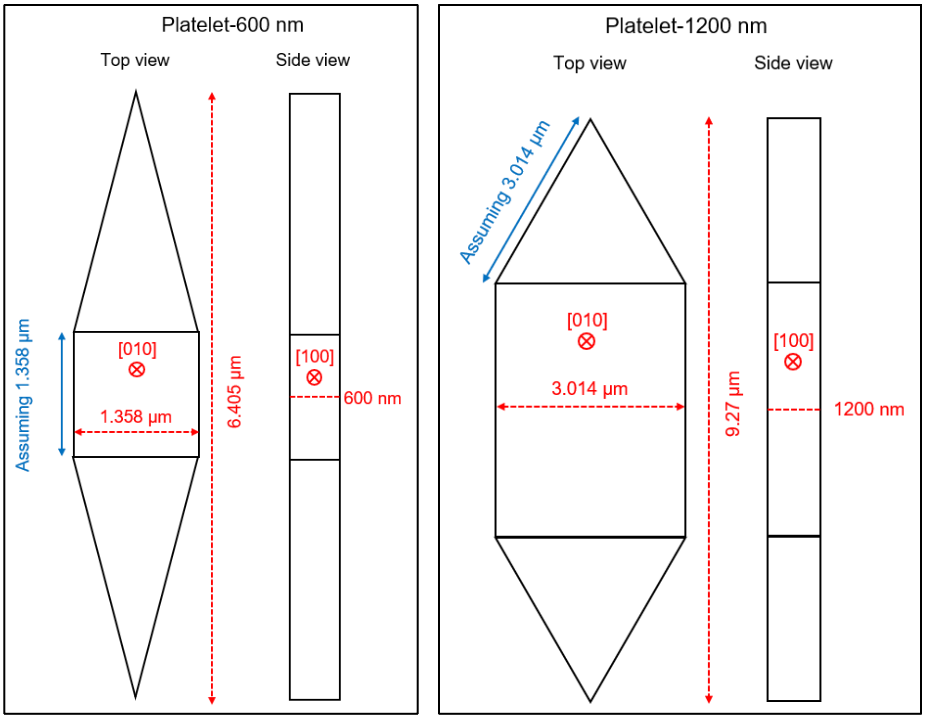

For Platelet-600 nm particles:

$$\frac{(010)}{[010]} = \frac{1.358 \times 2.5253 + 1.358 \times 1.358}{0.6} = 8.789\ \mu\text{m}$$

$$(010)\ \text{ratio} = \frac{(1.358 \times 2.5253 + 1.358 \times 1.358) \times 2}{[(1.358 \times 2.5253 + 1.358 \times 1.358) \times 2 + (2.615 \times 4 + 1.358 \times 2) \times 0.6]} = 0.572$$

For Platelet-1200 nm particles:

$$\frac{(010)}{[010]} = \frac{3.014 \times 4.05 + 3.014 \times 2.61}{1.2} = 16.7277\ \mu\text{m}$$

$$(010)\ \text{ratio} = \frac{(3.014 \times 4.05 + 3.014 \times 2.61) \times 2}{[(3.014 \times 4.05 + 3.014 \times 2.61) \times 2 + (3.014 \times 4 + 4.05 \times 2) \times 1.2]} = 0.624$$

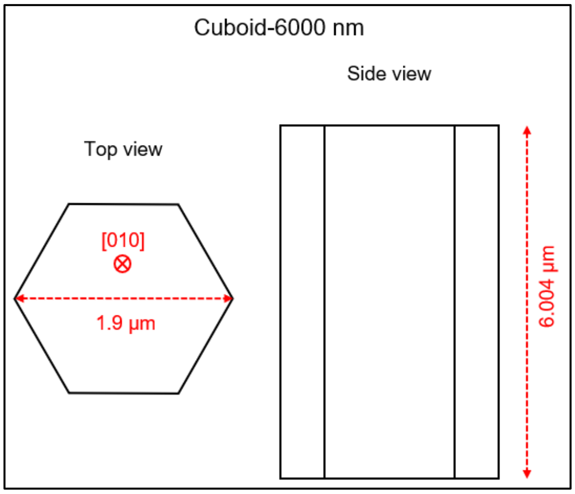

For Cuboid-6000 nm particles:

$$\frac{(010)}{[010]} = \frac{(0.95 + 0.95 \times 2) \times 0.8227}{6.004} = 0.391\ \mu\text{m}$$

$$(010)\ \text{ratio} = \frac{(0.95 + 0.95 \times 2) \times 0.8227 \times 2}{[(0.95 + 0.95 \times 2) \times 0.8227 \times 2 + 0.95 \times 6.004 \times 6]} = 0.1205$$

Supplementary References

1 Blöchl, P. E. Projector augmented-wave method. *Phys. Rev. B* **50**, 17953-17979, doi:10.1103/PhysRevB.50.17953 (1994).

2 Kresse, G. & Furthmüller, J. Efficient iterative schemes for ab initio total-energy calculations using a plane-wave basis set. *Phys. Rev. B* **54**, 11169-11186, doi:10.1103/PhysRevB.54.11169 (1996).

3 Kresse, G. & Furthmüller, J. Efficiency of ab-initio total energy calculations for metals and semiconductors using a plane-wave basis set. *Computational Materials Science* **6**, 15-50, doi:https://doi.org/10.1016/0927-0256(96)00008-0 (1996).

4 Perdew, J. P., Ernzerhof, M. & Burke, K. Rationale for mixing exact exchange with density functional approximations. *The Journal of Chemical Physics* **105**, 9982-9985, doi:10.1063/1.472933 (1996).

5 Perdew, J. P., Burke, K. & Ernzerhof, M. Generalized Gradient Approximation Made Simple. *Physical Review Letters* **77**, 3865-3868, doi:10.1103/PhysRevLett.77.3865 (1996).

6 Aykol, M., Kim, S. & Wolverton, C. van der Waals Interactions in Layered Lithium Cobalt Oxides. *The Journal of Physical Chemistry C* **119**, 19053-19058, doi:10.1021/acs.jpcc.5b06240 (2015).

7 Vladimir, I. A., Aryasetiawan, F. & Lichtenstein, A. I. First-principles calculations of the electronic structure and spectra of strongly correlated systems: the LDA+ U method. *Journal of Physics: Condensed Matter* **9**, 767, doi:10.1088/0953-8984/9/4/002 (1997).

8 Wang, L., Maxisch, T. & Ceder, G. Oxidation energies of transition metal oxides within the GGA+U framework. *Phys. Rev. B* **73**, 195107, doi:10.1103/PhysRevB.73.195107 (2006).

9 Jain, A. *et al.* Commentary: The Materials Project: A materials genome approach to accelerating materials innovation. *APL Materials* **1**, doi:10.1063/1.4812323 (2013).

10 Wang, L., Zhou, F., Meng, Y. S. & Ceder, G. First-principles study of surface properties of $LiFePO_4$: Surface energy, structure, Wulff shape, and surface redox potential. *Phys. Rev. B* **76**, 165435, doi:10.1103/PhysRevB.76.165435 (2007).

11 Yan, G. *et al.* The role of solid solutions in iron phosphate-based electrodes for selective electrochemical lithium extraction. *Nature Communications* **13**, 4579, doi:10.1038/s41467-022-32369-y (2022).

12 Abdellahi, A., Akyildiz, O., Malik, R., Thornton, K. & Ceder, G. Particle-size and morphology dependence of the preferred interface orientation in $LiFePO_4$ nano-particles. *Journal of Materials Chemistry A* **2**, 15437-15447 (2014).

13 Tasker, P. W. The stability of ionic crystal surfaces. *Journal of Physics C: Solid State Physics* **12**, 4977, doi:10.1088/0022-3719/12/22/036 (1979).

14 Wulff, G. XXV. Zur Frage der Geschwindigkeit des Wachsthums und der Auflösung der Krystallflächen. *Zeitschrift für Kristallographie - Crystalline Materials* **34**, 449 - 530 (1901).

15 Sun, W. & Ceder, G. Efficient creation and convergence of surface slabs. *Surface Science* **617**, 53-59 (2013).

16 Li, Y. *et al.* Fluid-enhanced surface diffusion controls intraparticle phase transformations. *Nat. Mater.* **17**, 915-922, doi:10.1038/s41563-018-0168-4 (2018).

17 Hong, L. *et al.* Two-dimensional lithium diffusion behavior and probable hybrid phase

transformation kinetics in olivine lithium iron phosphate. *Nature Communications* **8**, 13, doi:10.1038/s41467-017-01315-8 (2017).

18 Nan, C., Lu, J., Chen, C., Peng, Q. & Li, Y. Solvothermal synthesis of lithium iron phosphate nanoplates. *Journal of Materials Chemistry* **21**, 9994-9996, doi:10.1039/C0JM04126B (2011).
